# The risk status, signatures of adaptation, and environmental suitability of village-based indigenous chickens from certain regions of Limpopo and KwaZulu-Natal provinces of South Africa

**DOI:** 10.3389/fgene.2024.1450939

**Published:** 2024-12-18

**Authors:** Reneilwe Rose Mogano, Takalani Judas Mpofu, Bohani Mtileni, Khanyisile Hadebe

**Affiliations:** ^1^ Department of Animal Science, Tshwane University of Technology, Pretoria, South Africa; ^2^ Agricultural Research Council, Biotechnology Platform, Pretoria, South Africa

**Keywords:** redundancy analysis, habitat suitability, MaxEnt, village chickens, conservation strategies, effective population size

## Abstract

Indigenous chickens are an important Farm Animal Genetic Resource (FAnGR) in South Africa as they alleviate poverty and are a source of protein. Climate change and market demand for high-performing exotic breeds threaten and undermine locally adapted village chickens. The current study explored the risk status and signatures of adaptation of village-based indigenous chickens from two provinces and mapped their environmental suitability across the country. A total of 244 village chickens from rural areas of the Capricorn (n = 85) and Sekhukhune (n = 113) districts of Limpopo province; the Harry Gwala (n = 21) and uMzinyathi (n = 25) districts of KwaZulu-Natal province were genotyped using the Illumina 60K BeadChip. The conservation flock comprised Ovambo (OV; n = 10), Potchefstroom KoeKoek (PK; n = 20), and Venda (VD; n = 20). Naked Neck (NN; n = 20), New Hampshire (NH; n = 10), White Leghorn (WL; n = 10), and White Plymouth Rock (WR; n = 10) from the Agricultural Research Council Poultry Breeding Unit were used as reference populations and representative of flocks under conservation. The effective population size (*Ne*) in village chickens and conserved flocks ranged from 18 to 53 and 26 to 38 at 12 generations ago, respectively. PC1 and PC2 explained 5.64% of the total variation, which resulted in five clusters with the Venda, Naked Neck, and White Leghorn being separated from village chickens. The first three redundancy analysis (RDA) axes capture 46.8% of the total genetic variation used to detect significant outlier SNPs. A total of 386 outlier SNPs associated with all 10 environmental variables were detected. Using ecological niche modeling, chickens from Dipakakeng, Mgababa, and Podu villages, Limpopo, had a localized predicted suitability probability, while chickens originating from Nhlonga village, KwaZulu-Natal, had a broader distribution of predicted suitability habitats with elevation and BIO6 being important variables. The results of this study provide insight into the risk status, geographic suitability, and contributing environmental factors of indigenous chickens that can be used to influence conservation and improvement decisions.

## Introduction

In South Africa, as in most developing countries, indigenous chickens contribute significantly to the livelihood of rural communities (Mtileni et al., 2009; Malatji et al., 2016). Indigenous chicken production is not only a major source of income for rural communities but also serves as a significant supply of animal protein as well as a vital role in social, traditional, religious, and other customs (Mtileni et al., 2012; Nyoni and Masika, 2012; Yusuf et al., 2014; Gunya et al., 2020; Behura and Samal, 2022). Since their domestication, indigenous chickens have maintained a wide geographic spread across diverse agro-ecological zones (Hata et al., 2021; Lawal and Hanotte, 2021), including resistance to locally prevailing diseases and parasites (Gheyas et al., 2022), leading to their localized adaptation. Consequently, a relationship is expected between the phenotypic and genetic variation, prevailing environmental conditions, and other livestock dynamics such as the production system, originating location, etc. (Alemayehu and Getu, 2016). As an example, the naked neck, frizzle, silky, and dwarfism phenotypes have been reported to promote thermoregulation in hot and humid regions (Cahaner et al., 2008; Vandana et al., 2021). These phenotypes are found across South Africa (van Marle-Koster et al., 2001) and other African countries such as Nigeria (Manyelo et al., 2020), Ghana (Naazie, 2007), Kenya (Njenga, 2005), and Botswana (Machete et al., 2021a).

Environmental components such as high temperatures negatively impact chickens’ physiological response processes, such as oxidative stress, an acid-base imbalance, decreased immunity, and increased levels of corticosterone and cortisol (Lara and Rostagno, 2013; Onagbesan et al., 2023). These crucial physiological responses can lead to high mortality, reduced feed intake, reduced body weight, and a decrease in meat and egg quality (Wasti et al., 2020). They can also indirectly influence available resources such as food availability and quality as well as prevailing diseases (Kumari and Nath, 2018). The extensive nature of the village chicken production systems does not provide a buffer for the chickens as those in intense production systems, exposing them to high air temperatures and radiant heat from direct sun while scavenging outside during the day (Nyoni et al., 2022). Precipitation has been linked to the type of available crops and the quantity, as well as the availability of scavenging feed resources and edible soil fauna and diseases (Ncobela and Chimonyo, 2015; Zhang et al., 2016; Kebede et al., 2021; Bedane et al., 2022). Furthermore, elevation was found to impact the chicken’s physiological tolerance to ambient oxygen. For instance, these studies (Gou et al., 2007; Wei et al., 2007; Zhang et al., 2008) revealed that Tibetan chickens have adapted to high-altitude environments through physiological changes such as larger organs, lower oxygen partial pressure, and higher hemoglobin concentrations to enhance oxygen-carrying capacity, ultimately leading to their adaptation to high-altitude environments. Other factors, such as the soil properties like soil clay content, are an excellent indication of soil fertility and impact feed availability for scavenging chickens (Kome et al., 2019; Kebede et al., 2021), and therefore, can potentially contribute to the variations in the microbiome and animal response. Unfortunately, the contribution of these selection pressures to the variation we see today in chickens remains unknown (Nyoni et al., 2019).

The development and advancement of next-generation sequencing (NGS) technologies, remote sensing, and geographical information systems (GIS) (Kebede et al., 2021) have enabled the study of the genomic basis of adaptation (Storfer et al., 2018) as well as environmental factors contributing to the adaptive variation in indigenous chickens in the current study. In addition, by integrating ecological niche modeling, an understanding of the relationship between species distribution ranges and environmental variables can be gained to help predict the relative suitability of habitats for particular species (Warren and Seifert, 2011; Li et al., 2022). The use of microsatellite markers in indigenous livestock has been previously reported in the literature for indigenous chickens (Mtileni et al., 2011a; Fathi et al., 2017; Okumu et al., 2017; Habimana et al., 2020; Nxumalo et al., 2020; Rashid et al., 2020; Sabry et al., 2021; Ajibike et al., 2022; Zhuang et al., 2023), goats (Garritsen et al., 2015; Pakpahan et al., 2023), sheep (Peters et al., 2010), and cattle (van der Westhuizen et al., 2020). However, they are gradually being replaced by single nucleotide polymorphisms (SNPs) (van Marle-Köster and Visser, 2018). The first panel was developed in 2005 with a panel of 3072 SNPs (Muir et al., 2008). With continuous development, an Illumina Chicken iSelect 60K BeadChip array (Groenen et al., 2011) and an Affymetrix^®^ Axiom^®^ 600 K SNP array are available (Kranis et al., 2013).

These arrays have been used for genetic diversity analysis, breed relatedness analysis, genome-wide associate studies, quantitative character positioning analysis of quantitative trait loci, selective evolution investigation, and genomic selection (Groenen et al., 2011; Liu et al., 2019; Gonzalez-Prendes et al., 2022; Riggio et al., 2022; Xu et al., 2022). In Africa, there is little information on the utility of SNP arrays in exploring the adaptive variation of indigenous chickens, with most studies focusing on population structure and diversity (Khanyile et al., 2015; Banos et al., 2020; Walugembe et al., 2020; Machete et al., 2021a; Gheyas et al., 2022). In line with previous studies where comparisons between South African indigenous chickens from villages and conservation chicken populations highlighted differentiation (Khanyile et al., 2015; van Marle-Koster and Nel, 2000 Mtileni et al., 2016) revealed a genetic structure that can be exploited to identify drivers of the risk status and local adaptation in chicken populations. This comparison is justified as the conservation flocks (Venda, Ovambo, Potchefstroom KoeKoek, and Naked Neck) represent an intensive production system at the Agricultural Research Council–Poultry Breeding Unit, in contrast to village populations where no formal breeding structure or management is practiced. The aim of the study is to investigate the environmental suitability of indigenous chickens and estimate the risk status in different regions of KwaZulu-Natal and Limpopo provinces of South Africa. In the face of climate change, the study findings could be used to guide future conservation and improvement efforts through breeding for climate-resistant breeds in their intended environments.

## Materials and methods

### Chicken populations and sample collection

**FIGURE 1:**
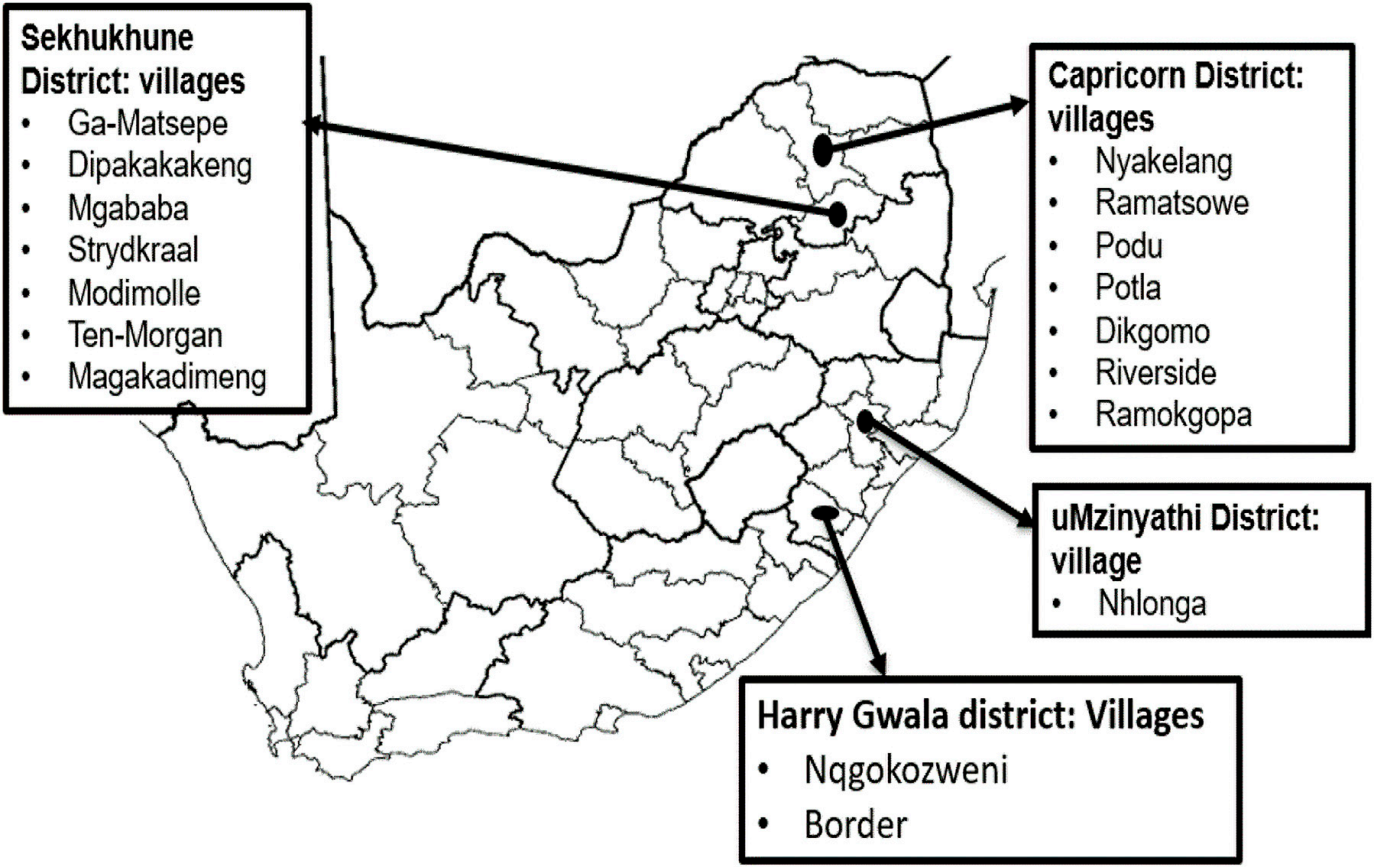
Sampling regions of South Africa.

A total of 244 chickens were randomly sampled from rural areas of the Capricorn (n = 85) and Sekhukhune (n = 113) districts of Limpopo province and the Harry Gwala (n = 21) and uMzinyathi (n = 25) districts of KwaZulu-Natal province (Figure 1). These animals were non-descript and were represented by a variety of phenotypic characteristics. They are termed “village chickens” to denote their originating production system. Sampled areas were chosen for the study based on their unique differences in climatic characteristics, production system potential, and the farmer’s willingness to participate in the community-based breeding program. The conservation flock: Ovambo (OV; n = 10), Potchefstroom KoeKoek (PK; n = 20), Venda (VD; n = 20), Naked Neck (NN; n = 20), New Hampshire (NH; n = 10), White Leghorn (WL; n = 10), and White Plymouth Rock (WR; n = 10) from the Agricultural Research Council, Poultry Breeding Unit were used to compare and unravel the effect of the production system and as reference populations for population structure of the village chickens. The conservation flocks are kept in an intensive production facility, ranging from 100 to 150 chickens (van Marle-Koster et al., 2008) with a low genetic variation (Khanyile et al., 2015). Blood samples were collected by brachial wing venepuncture onto the Whatman FTA cards (Whatman BioScience, UK) and transported to the laboratory at room temperature.

### DNA extraction and genotyping

Genomic DNA was extracted using the Macherey Nucleospin Tissue kit (MNT, MACHEREY-NAGEL) according to the manufacturer’s protocol. DNA quality was determined using 1% agarose gel and quantified using the NanoDrop instrument (NanoDrop Technologies, Wilmington, USA). Genotyping data were generated using the Illumina chicken iSelect SNP60K Bead chip using the Infinium assay compatible with the Illumina HiScan SQ genotyping platform at the Agricultural Research Council-Biotechnology Core Facility. PLINK (Purcell et al., 2007) was used for quality control based on the following criteria: individuals with a genotyping rate less than 95%, SNPs with a call rate less than 95%, SNPs with a minor allele frequency (MAF) ≤ 5% and those that deviated from Hardy–Weinberg equilibrium (HWE) p < 0.00001 were removed. A total of 244 individuals and 37,902 SNPs remained for further downstream analysis.

### Ecological niche modeling for suitability

Using the Quantum Geographic Information System (GQIS) software version 2.28.3, file layers were aligned and made to have the same extent and resolution before being transformed into acceptable ASCII raster files for species distribution modeling. MaxEnt version v3.3.3 (Phillips et al., 2006) was used for ecological niche modeling (ENM), and the predictive performance of the most important environmental variables was measured using test gain in MaxEnt v3.3.3 (Phillips et al., 2006). Only the village populations were considered for this analysis.

### Redundancy analysis (RDA) for the genotype-environmental association

Global positioning system (GPS) data were recorded for the sampled village chickens. Environmental data included climatic variables (n = 19), elevation, and solar radiation from the WorldClim database at a spatial resolution of 30-arc seconds (1 km^2^) based on mean values of 30 years (1970–2000) (Fick and Hijmans, 2017). Soil variables (n = 7) from the SoilGrid system at 250 m resolution (Hengl et al., 2017) were extracted using the raster R package (Hijmans R, 2023) (Supplementary Table S1). No vegetation data were extracted due to a lack of data in recorded GPS coordinates of sampling sites, and vegetation data are not included for further analysis.

To prevent overestimation and reduce model complexity, a correlation analysis was performed using ggcorr in the *GGally* R package (Schloerke et al., 2013) in R (R Development Core Team, 2019), and highly correlated variables (≥0.6) were removed. Redundancy analysis (RDA) was done using a *vegan* R package (Oksanen et al., 2022), and the threshold for significant SNPs was (SD ≥ 3).

### Gene annotation

Genes were retrieved using the *Ensembl BioMart* online tool (accessed 15 June 2024) (Smedley et al., 2009) using the chromosomal position, from a size of 1,000 bp of the SNP or region, depending on the analysis. The Ensembl Genes 100 database and the BioMart tool were also used to identify the corresponding orthologous chicken genes based on the GRCg6a reference genome.

The SNPs identified from the RDA were processed using the functional annotation tool implemented in the Database for Annotation, Visualization, and Integrated Discovery (DAVID) gene ontology and annotation tool for gene enrichment analysis (Huang et al., 2009).

### Population structure

The principle component analysis was done using adegenet (Jombart, 2008), an R package to assess the population structure, and the admixture analysis was done using the R package LEA (Frichot and Francois, 2015) to assess the most probable number of ancestral populations based on the SNP genotype data.

Admixture analysis estimates individual ancestries by efficiently computing maximum likelihood estimates in a parametric model (Alexander and Lange, 2011). The R package LEA (Frichot and Francois, 2015) was used to assess the most probable number of ancestral populations based on the SNP genotype data. The optimal number of clusters (K-value) was determined as the lowest cross-entropy validation (CV error). The cross-entropy criterion was used to determine the number of ancestral populations.

The extent of linkage between pairs of SNPs between a chromosome was assessed using pairwise r2 estimation in conservation flock and village chickens. The r2 estimates are defined as the squared correlation coefficient of alleles at two loci (Lu et al., 2012). Calculation is based on two loci, A and B, with each locus having two alleles. The frequency of the haplotypes is denoted as F11, F12, F21, and F22, respectively, for haplotypes A1B1, A1B2, and A2B2, and as FA1, FA2, FB1, and FB2 for alleles A1, A2, B1, and B2 (Machete et al., 2021b). By default, PLINK only reports r2-values above 0.2. To allow reporting of all r2-values observed in the populations, the–r2–window-ld 0 option was used. An additional option, –r2 –window-snp 2000 –kb 10,000, allowed for the estimation of r2 for SNP marker pairs.

### Effective population size

Effective population size (*Ne*) was estimated on the known relationship between *r* ^2^, *Ne,* and the recombination rate (c) between two loci. Linkage disequilibrium (LD) makes it possible to estimate effective population size (*N*_*e*_) using the following equation (Sved, 1971):

$$E\;\left(r^{2}\right)=\frac{1+1}{\left(\alpha +\text{KNeC}\right)n}.$$

r^2^ is the LD between different makers, *N*_*e*_ is the effective population size, c is the genetic distance between various markers measured, n is the individual experimental sample size, α = 1 in the absence of mutation, α = 2 if the mutation is present, k = 4 for auto chromosomes, and k = 2 for an x chromosome. This was implemented in *SNeP* software (Barbato et al., 2015).

## Results

### Environmental suitability

**FIGURE 2:**
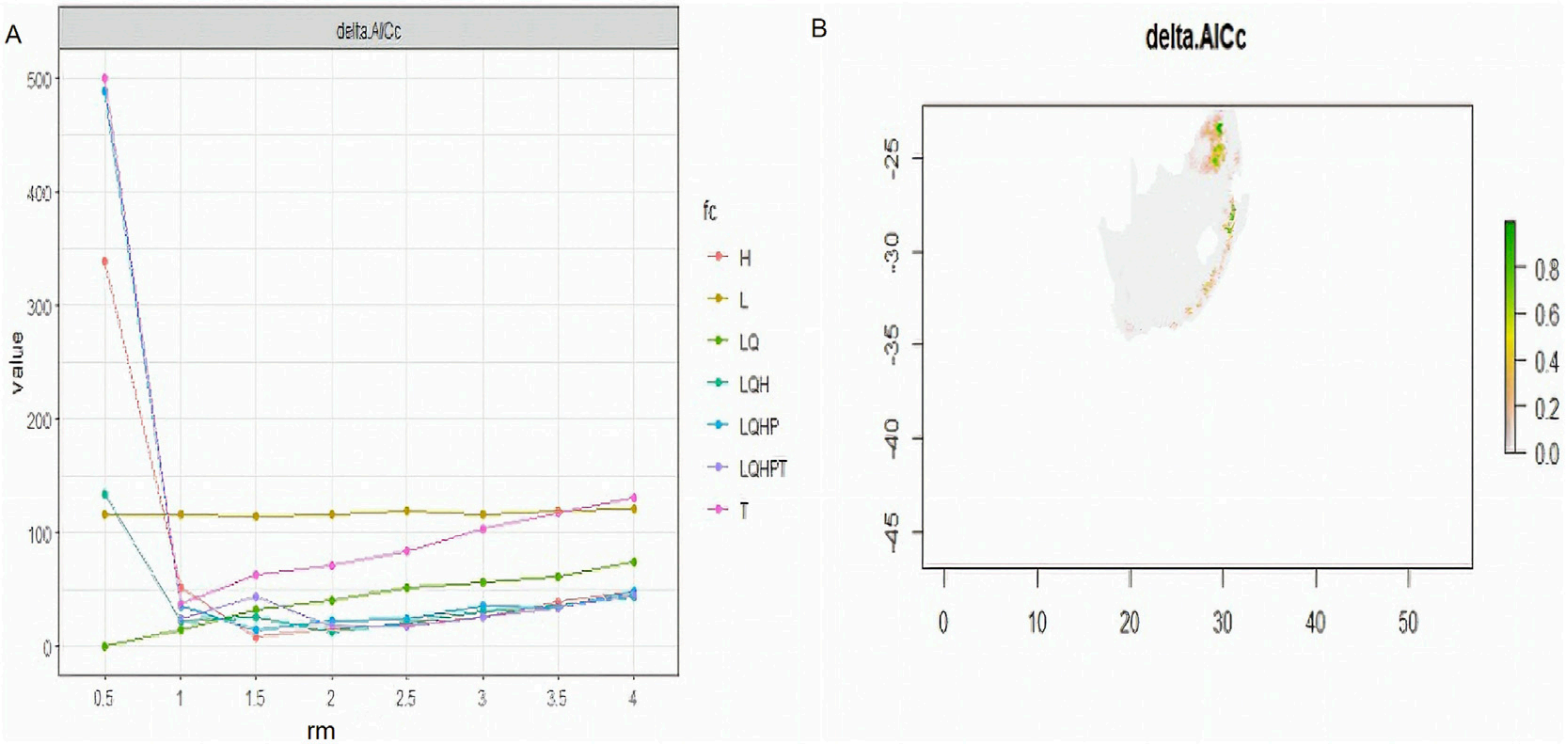
AICc values for analysed feature combination, with a map produced using a best optimum parameter (LQ and RM = 0.5).

**FIGURE 3:**
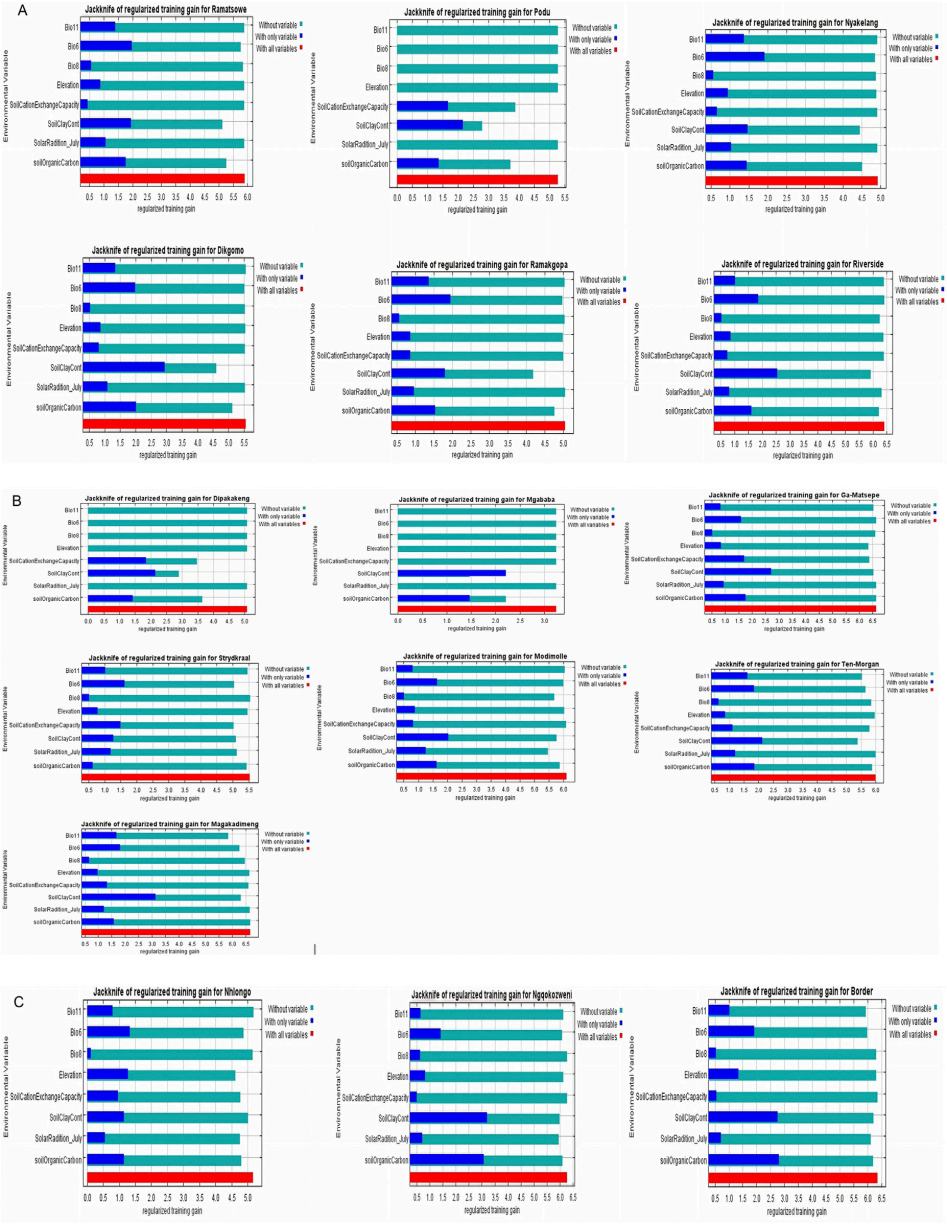
Jack-knife regularization training test of villages in KwaZulu-Natal and Limpopo Provinces.

MaxEnt offers six feature classes for reducing overfitting: linear, quadratic, product, hinge, threshold, and categorical. The ENMeval identified LQ (linear and quadratic feature classes) feature combination with regularization multiplier = 0.5 as the best parameter (Figure 2). This had the lowest AICc delta value and was selected by MaxEnt to produce suitability maps. The species distribution model maps at the village level illustrate ideal environmental conditions. The variable’s significance was determined using the Jack-knife test (Figures 3A–C).

**FIGURE 4:**
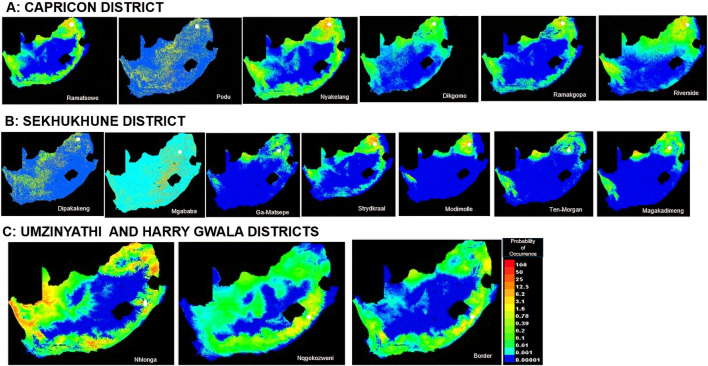
Suitability map at village level.

In Limpopo, the soil clay content was only most significant when used in isolation and decreased gain when excluded from the model compared to other variables, resulting in soil clay content being the only variable with significant importance in mapping suitability maps for the following villages: Dipakakeng, Mgababa, Ten-Morgan, Ramokgopa, Riverside, Podu, Dikgomo, and Riverside. In Strydkraal village, BIO6 had the most importance when used in isolation and decreased gain when excluded; for Modimolle village, solar radiation decreased gain when excluded, and in Magakadimeng village, BIO11 decreased gain when excluded. For KwaZulu-Natal, BIO6 had the most importance, and elevation decreased gain when excluded for Nhlonga village. For Nqgokozweni village, soil clay content had the most importance when used in isolation, and solar radiation decreased gain when excluded, soil organic carbon had the most importance when used in isolation, and BIO6 decreased gain for Border village (Figures 4A–C). Chickens from Nhlonga village had a broader distribution in potentially suitable environments, ranging to the Northern Cape, Limpopo, and Eastern Cape provinces. The chickens from Dipakakeng, Mgababa, and Podu villages had a low predicted suitability probability, with soil clay content being the only variable that had significant importance when used in isolation and also when omitted from the model.

### Environmental association

**FIGURE 5:**
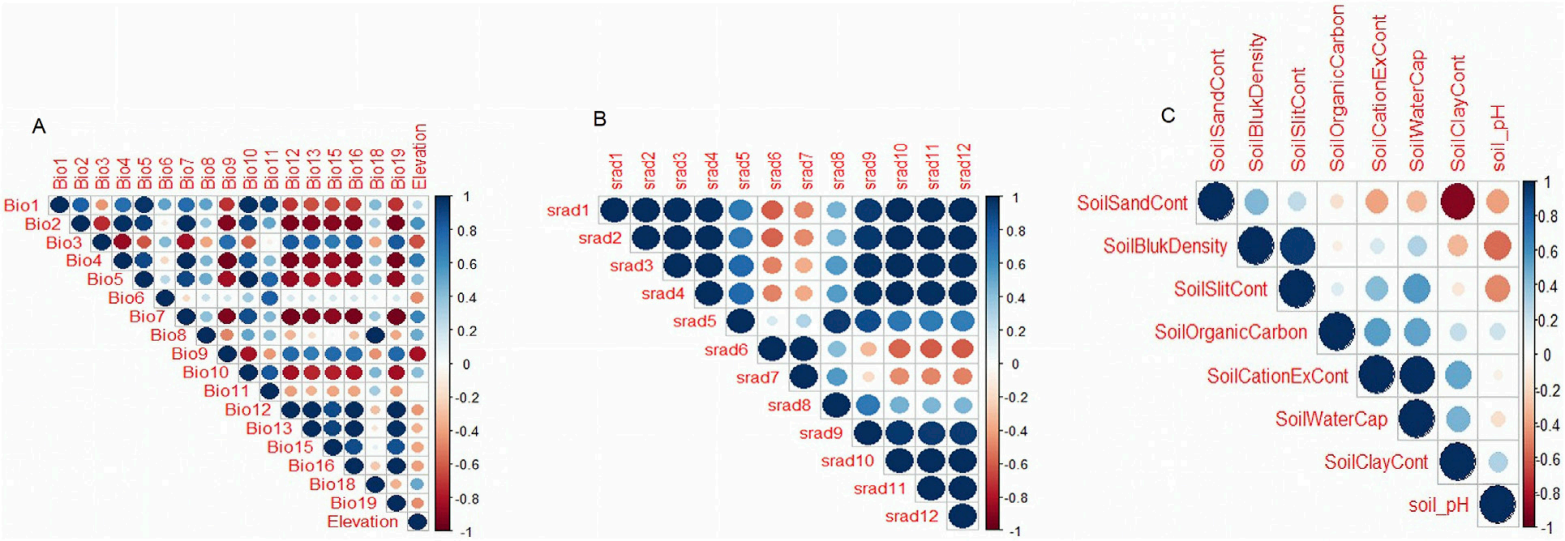
Correlation test of environmental variables.

A correlation test was done on environmental variables to remove highly correlated variables by using a threshold of ≥0.6 (Figure 5), and only 10 variables remained for downstream analysis. These variables are the minimum temperatures of the coldest month (BIO6), minimum temperature of the wettest quarter (BIO8), mean temperature of the coldest quarter (BIO11), elevation, soil cation exchange capacity, soil clay content, soil organic carbon content, soil pH, soil silt content, and solar radiation in the seventh month.

**FIGURE 6:**
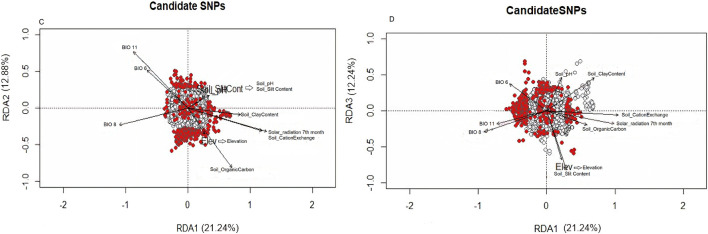
**(A)** RDA loadings, **(B, C)** Significant candidate SNPs related with environmental variables RDA1 and RDA2; **(D)** Significant candidate SNPs related with environmental variables RDA1 and RDA3.

All village chickens were distributed in RDA space with no clear clustering, reflecting their relationship with the ordination axes, which are linear combinations of the predictor variables (Figure 6B). The first three RDA axes were significant, and these together accounted for 46.36% of the total captured variance. They were used to determine SNPs showing significant association with environments (Figure 6A). SNPs that exceeded the standard deviation (SD > 3) in the rear end of the SNP loadings were denoted as outlier SNPs. A total of 386 outliers were identified and differentiated following the correlation of environmental predictors (Supplementary Appendix Table S2). RDA1 explained a variance of 21.24% (Figure 6C). Outlier SNPs on RDA 1 represented multi-locus sets of SNPs associated with solar radiation and soil cation exchange content. RDA 2 explained a variance of 12.88% and represented genotypes associated with BIO11 and soil organic carbon content. RDA 3 explained a variance of 12.24% (Figure 6D) and represented genotypes associated with elevation and soil slit content. BIO11 was associated with 127 SNPs, BIO6 was associated with 60 SNPs, soil carbon exchange content was associated with 45 SNPs, and solar radiation in the seventh month was associated with 44 SNPs. The first 30 genes with a high correlation (r = 0.3–0.6) (listed in Supplementary Table S2) were closely examined to understand their biological functions.

The *ND2* gene (r = 0.48) located on chromosome 10 is involved in metabolic pathways and has been linked to soil cation exchange. The *UXS1* gene (r = 0.44) located on chromosome 1 is involved in the biosynthesis of nucleotide sugar pathways and has been linked to BIO6. The *ERBB4* gene (r = 0.44) located on chromosome 7 is involved in the MAPK signaling pathway and has been linked to BIO11. The *NRG2* gene (r = 0.48) located on chromosome 13 is involved in the ErbB signaling pathway and has been linked to elevation. *MYO18B* gene (r = 0.43) is located on chromosome 15, is involved in motor protein pathways, and has been linked to soil slit content. The *AGRN* gene (r = 0.41) located on chromosome 21 is involved in ECM-receptor interaction pathways and has been linked to BIO6. The following gene pathways with a high correlation were not identified, implying that they are not annotated. These genes include *TLCD4, ADAMTS2, MUC2, SLC9A9, LMTK2, LRBA, ADGRL, KHDRBS3, WDR89, EPHA4, NPM1, and GJBS*.

### Population structure analysis

**FIGURE 7:**
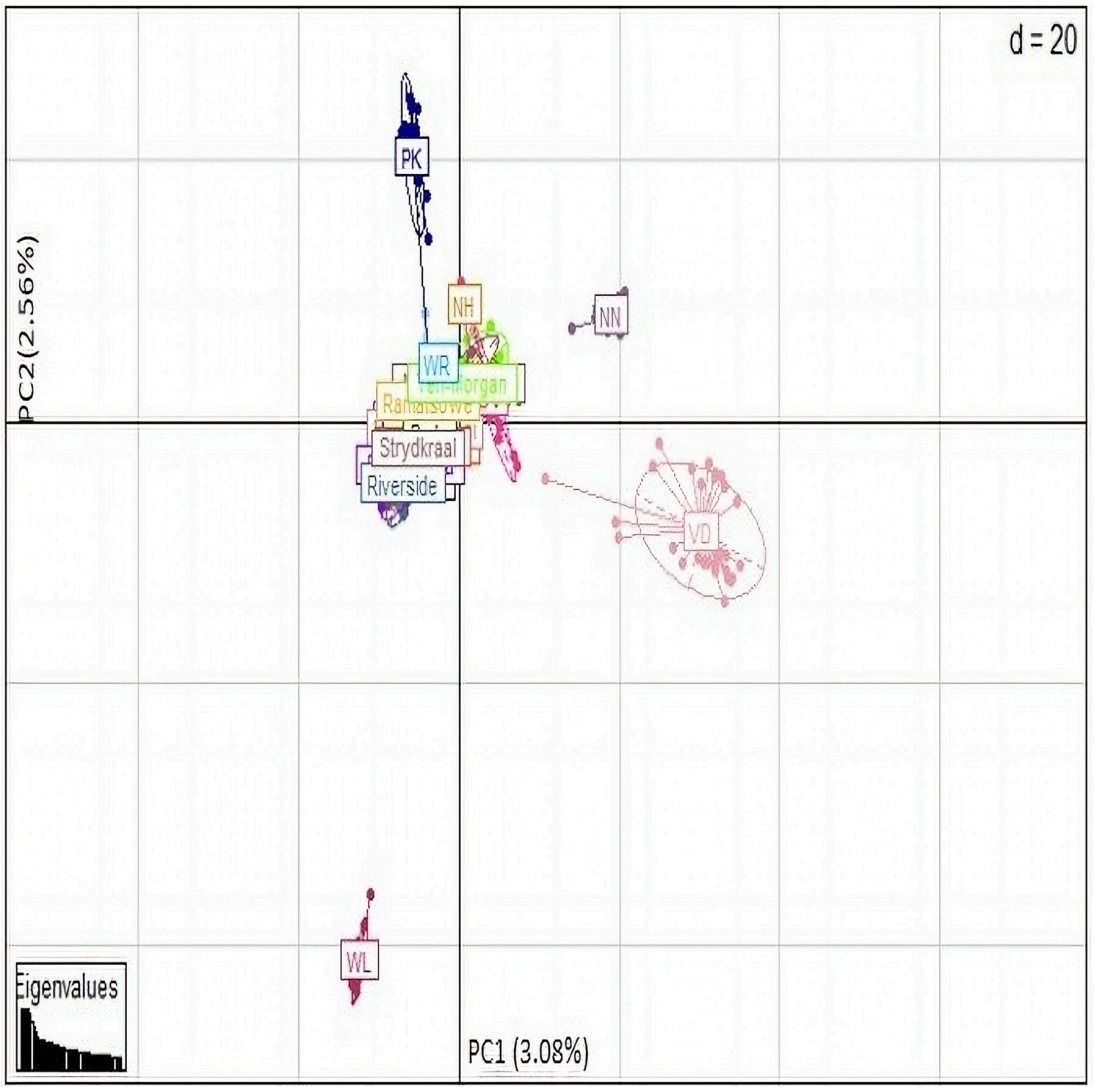
PCA base clustering plot.

Principal component analyses were performed to explore and identify the genetic clustering of the village chickens (Figure 7). The first principle component explained 3.08% of the total variation and separated the Naked Neck (NN), Venda (VD), and White Leghorn (WL) conservation breeds from the village chickens. The second principal component explained 2.56% of the variation and genetically clustered the Ovambo (OV), exotic multi-purpose New Hampshire (NH), and White Plymouth Rock (WR) breeds with the village chickens.

**FIGURE 8:**
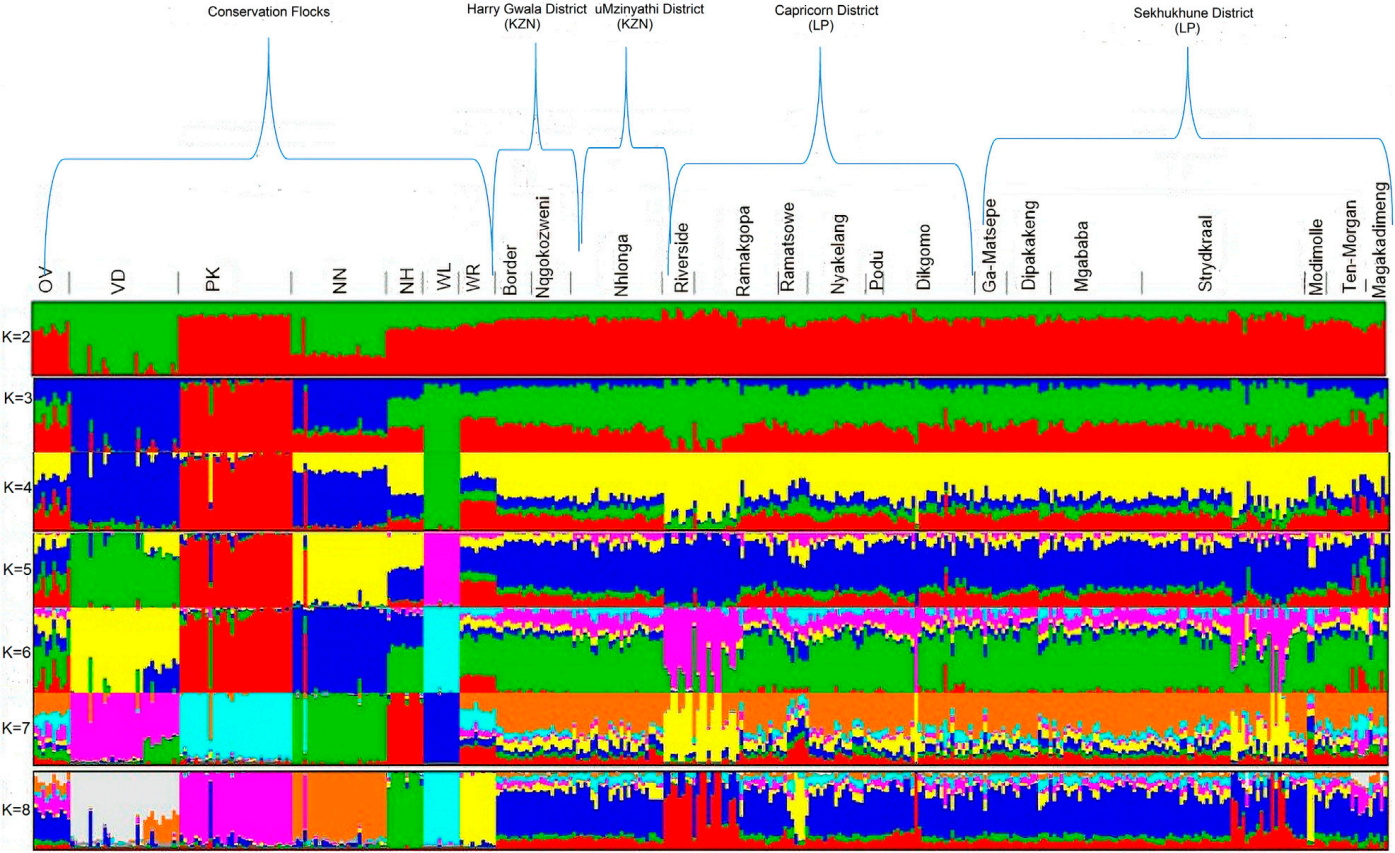
Admixture population structuring.

The optimal K-value (0.87) for admixture was K = 5 (Figure 8). Membership coefficients (K = 2 to K = 8) for the study populations are illustrated in Figure 8. At K = 2, the VD and NN had different genetic backgrounds compared to others. At K = 3, the WL breed separated from both village chickens and conservation flocks, indicating a low diversity within this population. At K = 4, the OV, NH, and WR had similar frequency genetic backgrounds as the village-based chickens compared with other breeds. At K = 5, WL, NH, VD, PK, and NN formed five distinct clusters from village chicken populations; OV and WR had a slightly similar genetic variation with village chickens. At K = 8, OV and WR separated from village chickens. All conservation flocks formed independent clusters; Riverside, Ramatsowe, Dikgomo, and Magakadimeng held individuals with unique genetic lineage from the village chicken population.

### Linkage disequilibrium

The summarized estimates of the *r2* values for each chromosome of village chickens and conservation flocks of South Africa are illustrated in Supplementary Tables S2, S3. The LD was high in Ramatsowe, Modimolle, Magakadimeng, and Podu compared to other village chickens. In conservation flocks, New Hampshire had a high LD compared to other conservation flocks. A high LD in Ramatsowe was observed in chromosomes 6 and 7. In Podu village, chicken LD was high at chromosome 28, and for Modimolle and Magakadimeng, chromosome 16 had a high LD.

### Estimated effective population size (*Ne*)

**FIGURE 9:**
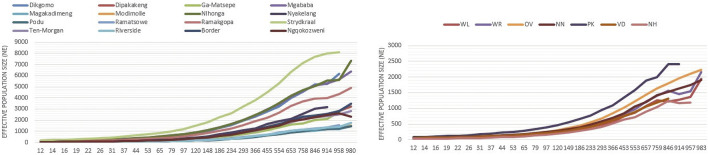
Trends of effective population size for indigenous chicken and conservation flocks.

Figure 9 shows the estimated *Ne* for village-based indigenous chickens and conservation chicken flocks 983 generations ago. Within the villages, chickens from Nhlonga and Strydkraal had the highest estimated *Ne*, which is above 4,000 *Ne* at 983 generations ago, and there was a constant decrease of *Ne* until village chickens from Limpopo province had a *Ne* of more than 135, and chickens in KwaZulu-Natal had a *Ne* of 303 at 97 generations ago. Furthermore, the lowest *Ne* in both village chickens and conservation flocks was at 12 generations ago. For village chickens, *Ne* ranged from 18–53, and for conservation flocks, it ranged from 26–38.

## Discussion

Understanding adaptive genetic variation has become critical in conservation strategies of adaptive biodiversity and breeding improvements. In this way, adaptability to various habitats can be explained by looking at breed origins, where environmental and human-caused environmental selection pressures have influenced their adaptation to various production constraints (Lozano-Jaramillo et al., 2019). The current study aimed to unravel the risk factors and signatures of the adaptation of village-based indigenous chickens from two provinces and map their environmental suitability across the country.

The failure to recognize the adaptive diversity of indigenous chickens has led to the introduction of exotic breeds due to their low productivity (Gebru et al., 2022), as the market favors the use of highly productive chicken breeds (Granevitze et al., 2007). The promotion of locally adapted breeds lies in designing appropriate management strategies for indigenous chickens and minimizing genetic dilution and erosion, especially in the face of climate change (Mtileni et al., 2016). Therefore, understanding the mechanism and drivers of local adaptation is crucial for breeding and developing climate-resilient flocks.

Ecological niche modeling has the flexibility to include any number of environmental factors in the evaluation process (Phillips et al., 2006) and captures the gene–environment interaction in shaping the livestock genome (Vallejo-Trujillo et al., 2021). MaxEnt uses data to relate environmental variables and occurrence points to calculate the likelihood of probable geographical suitability (Phillips et al., 2006). Soil is a categorical variable, indicating that soil type characteristics are not the same throughout the country, resulting in poor prediction of regions that are ideal for Podu village chickens. Clay, which consists of aluminosilicate minerals, is an important part of the soil (Abdeldjouad et al., 2019). Through their physical and chemical properties, clay minerals can be expected to have more nutrient reserves in tropical areas (Kome et al., 2019). Other soil properties, such as a high soil organic carbon environment, are distinguished by the presence of numerous earth-dwelling animals, such as insects and worms, which are good protein-rich food sources for chickens (Gheyas et al., 2021). In the current study, BIO6 and soil clay content were the most important environmental variables for mapping suitable maps for village chickens from the Limpopo province, while the BIO6, soil organic carbon, and soil clay content were significant in KwaZulu-Natal. Chickens from the Nhlonga village in KwaZulu-Natal had a broader suitability distribution for existence and fitness, covering the Northern Cape, Limpopo, and Eastern Cape provinces. This implies that village chickens from the Nhlonga village will be able to adapt to these areas and reach their peak levels of productivity and fitness. The province of KwaZulu-Natal experiences temperature increases, moderate winters, and extreme weather events such as droughts and floods (Connolly-Boutin and Smit, 2016; Leonard, 2022). The predicted regions show similar climatic conditions as KwaZulu-Natal, with Northern Cape province experiencing hot summers and drought and Limpopo experiencing frequent drought and high temperatures (Maponya, 2012; Maposa et al., 2021; Bahta and Musara, 2023). In the Limpopo province, chickens from Dipakakeng, Mgababa, and Podu villages were observed to have intermediate to poor predicted suitability across South Africa. These findings suggest tolerance to local environmental stressors.

According to the redundancy analysis (RDA), the highest environmental contributors were BIO11, BIO6, and soil organic carbon and solar radiation in the seventh month, with 127, 60, 45, and 44 SNP outliers identified, respectively. The genes TENM2, JARID2, and *TRAF3IP3* were associated with BIO6. The *TENM2* gene (r = 0.36) has been identified as a gene under putative selection in local chicken populations of Rwanda and Uganda (Fleming et al., 2016). This gene is involved in neural development and regulates the establishment of proper connectivity within the nervous system (Fleming et al., 2016; Del Toro et al., 2020). Calcium functions of *TENM2* may help activate *PRKCA* when stressors appear by stimulating calcium release (Fleming et al., 2016). In our study, this gene is observed to be involved in the ability of chickens to withstand cold months through various adaptive responses, such as shivering, for heat production and thyroid hormone release, which increases heat production in peripheral organs (Ouchi et al., 2022). The JARID2 gene (r = 0.47) is crucial to embryonic development, including heart and liver development, neural tube fusion, and hematopoiesis (Bateman et al., 2023). This gene may allow the chick to survive in the early stage of growth and fight infections. This gene was found to be related to antibody response to Newcastle disease in Rwanda chickens (Habimana et al., 2021). Kharrati-Koopaee et al. (2021) reported that the *JARID2* gene can help with the hypoxia adaptation process by binding to chromatin. Given that KwaZulu-Natal has the highest elevation of all provinces, at 3.451 m, this may be favorable for the village chickens from KwaZulu-Natal. The *TRAF3IP3* gene (r = 0.39), which aids in cell maturation, tissue development, and immune response, is expressed in the immune system (Li et al., 2018). It promotes immunological homeostasis and prevents innate immunity from becoming excessively aggressive by promoting TBK1’s ‘Lys-48′-dependent ubiquitination (Bateman et al., 2023). This gene was also found to have a good potential for *Salmonella* resistance in commercial layers (Psifidi et al., 2018).

Precipitation was associated with BIO8, which has an impact on the physiology or behavior of chickens. For example, excessive rainfall could trigger the spread of an infection, impair immunity, or cause dietary changes. The *FHIT* gene (r = 0.33) is associated with body mass index and may be related to carcinogenesis (Joannes et al., 2014; Ahmad et al., 2016). This gene appears in the chicken brain, colon, heart, kidney, liver, lung, spleen, and testis (Graczyk et al., 2017) and can act as a modulator of the stress response (Karras et al., 2014). It is part of a purine metabolism pathway in primordial germ cells that produce adenosine diphosphate (Rengaraj et al., 2013). The *MUC2* gene (r = 0.51) is responsible for lubricating and protecting the external surface of the internal epithelium tissue (Bateman et al., 2023). This gene is highly expressed in the gastrointestinal tract with a minimal appearance in the crop, ventricular, and brain of chickens (Jiang et al., 2013). It is a valuable gene for innate immune response to different pathogens (Jiang et al., 2013).

The genes *OPCML*, COL4A3, and *MFSD12* are associated with BIO11. The *OPCML* gene (r = 0.47) is found to be related to body weight at 12 weeks of age in chickens (Gu et al., 2011), and this gene was also found to be related to immune and cytokines in chickens (Yuan et al., 2015). This may help chickens fight off diseases or become resistant to diseases and survive the early stages of embryonic development. The COL4A3 gene (r = 0.39) plays an important role in the structural maintenance and normal development of skeletal muscle. The *MFSD12* gene (r = 0.32) is engaged in feather pigmentation by regulating melanogenesis gene expression, providing melanogenesis sites, controlling melanoblast migration, and transmitting melanogenesis substrates (Adelmann et al., 2020). This gene may be crucial for adaptation in chickens, as it protects their skin from harmful radiation due to their extensive scavenging.

The genes OPN3 and FGF20 are associated with solar radiation in the seventh month. The *OPN3* gene (r = 0.31) is associated with light-independent functions such as regulating melanogenesis and apoptosis in epidermal melanocytes and may be involved in the photorelaxation of airway smooth muscle cells (Ozdeslik et al., 2019). Opsins are photosensitive pigments that form a light-sensitive G protein-coupled receptor with retinaldehyde, allowing them to detect specific wavelength lines (Rios et al., 2019). This gene was found to be intact in the chicken’s retina (Kato et al., 2016). Birds use UVS to recognize coloration patterns, communicate with one another, hunt, flee, locate feed, and select a mate (Bennett et al., 1996). The *FGF20* gene (r = 0.34) is expressed during the early stages of feather placode patterning (Perini et al., 2021). *FGF20* signaling plays a crucial role in promoting cell condensation and feather primordium development in chickens (Song et al., 2004). There is diffuse expression of FGF receptors in the ectoderm and dermis before feather buds form (Yang et al., 2019). FGF20 is believed to be crucial for vertebrate skin development, suggesting that it could be incorporated into crossbreeding to create featherless chicken lines less susceptible to high temperatures (Wells et al., 2012).

Elevation was associated with the *ADGRL3* gene (r = 4.0), a member of the latrophilin subfamily of G protein-coupled receptors that are highly expressed in the brain, specifically in the amygdala, caudate, pontine nucleus, and cerebellum (Arcos-Burgos et al., 2019). The gene has been linked to the development of glutamatergic synapses and cell-to-cell signaling (Dalla Vecchia et al., 2019) and is said to function by interacting with leucine-rich repeat transmembrane proteins of fibronectin, particularly *FLRT2* and *FLRT3* (Kordon et al., 2023). Its function is linked to animal neuronal functioning (Ahmad et al., 2023). This can result in neuroplasticity, which is the capacity of the brain to reorganize its connections, structure, or function in response to external or internal stimuli, resulting in changes to its morphology and physiology (Marzola et al., 2023). It plays a significant role in hearing, memory, and feeling pain, etc. (Voss et al., 2017). The NRG2 gene (0.48) was also associated with elevation; it is involved primarily in the development of the nervous and cardiovascular systems (Mei and Nave, 2014). The primary receptor for NRG2 is HER4 (Sweeney et al., 2001). NRG2 plays an important role in the development of regional networks in the central nervous system, and the loss of NRG2 may lead to behavioral abnormalities in animals similar to those observed in human psychiatric disorders (Yan et al., 2018).

The ND2 gene (r = 0.48), found to be associated with soil cation exchange, participates in the mitochondrial respiratory chain and oxidative phosphorylation involved in energy metabolism (Barker et al., 2012; Yang et al., 2020). It is a subunit of *NADH* dehydrogenase, and the types of dietary fat and age have an impact on its manifestation (Lu et al., 2016; Zhang et al., 2016). According to a report, the production of reactive oxygen species in Tibetan chicken was substantially correlated with missense substitution in the *ND2* (Wang et al., 2013). This gene is crucial for cold climate adaptation, influencing mitochondrial coupling efficiency and ATP synthesis efficiency ratio coordination and aiding in climate adaptation (Gershoni et al., 2014). The *GPNMB* gene (r = 0.32) was associated with soil organic carbon. It is released into the bloodstream to perform its function under the hydrolysis of integrin metalloproteinase 10 (Rose et al., 2010). *GPNMB* is found to play a crucial role in various biological processes, such as melanin deposition (Zhang et al., 2013). It is found in all phases of melanosome formation (I–IV) but is particularly abundant in mature melanosomes (stages III and IV) (Hoashi et al., 2010). It gives color to the plumage of chickens, which is a vital trait as it influences consumer preferences and helps chickens camouflage themselves as defense mechanisms in their environment (Li et al., 2023). The *GPNMB* acts as a regulator of inflammation (Ripoll et al., 2007; Zhou et al., 2017). The *GPNMB* functions to reduce inflammation by increasing anti-inflammatory cytokines like IL10 and decreasing proinflammatory cytokines like TNFα, IL-6, and IL-12 (Saade et al., 2021), which aid in response to lesions in the tissue or infection (Saade et al., 2021), and can be triggered by pathogens that are present in their environment.

Both the PCA and admixture analysis clusters suggest a high degree of within-population variation between the village populations despite the wide geographic distribution. The Ovambo, New Hampshire, and White Plymouth Rock breeds had genetic backgrounds similar to those of the village populations. This could be attributed to the introduction and continuous use of New Hampshire and White Plymouth Rock in small-scale chicken production, which contributed to the admixture seen in these breeds. The White Plymouth Rock breed was introduced in South Africa during the era of African colonization and later was introduced in the conservation program, resulting in extensive mixing with local chicken lines (van Marle-Koster and Nel, 2000; van Marle-Koster et al., 2008). New Hampshire is one of the dual-purpose breeds that is kept in similar systems with the four conservation flocks. This breed has been considered as a breed for upgrading and developing local lines (van Marle-Koster et al., 2008). Ovambo chickens were popular and chosen by smallholder farmers due to their aggressiveness and dark plumage color (Tarwireyi and Fanadzo, 2013). This explains the clustering with village chickens, for they still maintained some similarity in allele frequency with village chickens. A previous study by Khanyile et al. (2015) found that Ovambo had a slight genetic similarity with village chickens and clustered with them. There is low diversity in the Naked Neck and Venda, indicating a loss of genetic components from their founder populations. The studies of Mtileni et al. (2011a) and Khanyile et al. (2015) showed that conservation flocks have diverged from their founder village chicken populations. The intensive production system, low number of founder individuals, and genetic isolation have been reported to lead to inbreeding depression (Charlesworth and Willis, 2009) and the founder effect (Mtileni et al., 2016).

The overall LD values between village chickens and conserved flocks were significantly different. The high value of LD in chromosome 16 was reported in these studies (Khanyile et al., 2015; Machete et al., 2021a). Khanyile et al. (2015) reported higher LD values in chromosome 16 for both village chickens and conservation flocks. A recent study by Machete et al. (2021a) also reported a high LD in chromosome 16 for Tswana chickens and Naked Neck. In our study, a high LD in chromosome 16 was observed in Modimolle and Magakadimeng village chickens. In addition, Khanyile et al. (2015) point out that evolutionary processes influencing LD work differently on different chromosomes and strains. Strydkraal village chickens had the lowest LD value when compared to other village chickens. This implies that they had the highest effective population size, which is supported by the estimation trends of effective population size analysis.

In conservation, the effective population size (*Ne*) is important because it affects genetic drift, inbreeding, and genetic diversity loss (Ryman et al., 2019). Franklin (1980) initiated the 50/500 rule that indicates populations with an inbreeding effective population size (*Nef*) of less than 50 are at risk of extinction. In this study, a low *Ne* was observed 12 generations ago, with village chickens having a *Ne* ranging from 18 to 53 and a conserved flock having a *Ne* ranging from 26 to 38. Low *Ne* levels can be caused by small founder animals and poor management of diversity within the breeding flock, which may, over time, lead to decreased genetic variability and genetic drift (Mtileni et al., 2016). Khanyile et al. (2015) indicated a low *Ne* of 49–57 in the village chickens and of 31–50 in the conservation flocks 97 generations ago, while in the current study, village chickens from Limpopo province had a *Ne* of more than 135 and 303 in KwaZulu-Natal at 97 generations ago. These *Ne* results of the study are higher than those of Khanyile et al. (2015). This difference may be due to good breeding practices and management (Bettridge et al., 2018), with no effect of population bottleneck and no overlap in generations that can lead to inbreeding. For instance, farmers cull old chickens with poor reproductive performance (Moges, Mellesse, and Dessie, 2010; Nyoni and Masika, 2012). In addition, there is high gene flow due to the migration of chickens to neighboring villages (Muchadeyi et al., 2007), increasing *Ne.* Zhang et al. (2023) found *ex situ* conserved Chinese chicken populations maintained in controlled environments retain less genetic diversity than *in situ* conserved populations.

## Conclusion

The study demonstrates the integration of ecological niche modeling and the use of genomic data to assess the current situation and prepare for future conservation programs for indigenous livestock species such as chickens. The study findings can aid in identifying suitable habitats and conservation hot spots as well as unravel genomic regions involved in environmental adaptation. These findings can be used to guide future planning and design of conservation programs to ensure that the indigenous chicken genetic resource is sustained. Management of the population size and improvements of current breeding and conservation programs to meet fitness needs can guarantee optimal production performance through climate-resilient breeding.

## Data Availability

All data is available in supplementary material and further inquiries can be directed to the corresponding authors.
