# Supplementary material for: The risk status, signatures of adaptation, and environmental suitability of village-based indigenous chickens from certain regions of Limpopo and KwaZulu-Natal provinces of South Africa
Source: Front Genet. 2024 Dec 18;15:1450939. doi: 10.3389/fgene.2024.1450939 (PMC11688331; doi:10.3389/fgene.2024.1450939)
Supplement: Supplementary file 1 [file DataSheet2.pdf]

Supplementary material

Table 2 Average r^2 values per chromosome and village chickens

Chr	Border	Nhlonga	Nqgokozweni	Nyakelang	Riverside	Ramatsowe	Ramokgopa	Podu	Dikgomo
1	0.12±0.15	0.07±0.09	0.14±0.17	0.11±0.14	0.24±0.25	0.26±0.27	0.08±0.09	0.28±0.27	0.06±0.08
2	0.13±0.15	0.07±0.10	0.14±0.17	0.11±0.14	0.24±0.24	0.29±0.28	0.08±0.09	0.27±0.26	0.06±0.08
3	0.13±0.15	0.07±0.09	0.13±0.16	0.12±0.14	0.27±0.26	0.25±0.26	0.07±0.09	0.26±0.26	0.07±0.09
4	0.13±0.15	0.07±0.10	0.15±0.17	0.11±0.13	0.26±0.25	0.24±0.25	0.07±0.09	0.26±0.26	0.07±0.09
5	0.12±0.14	0.07±0.10	0.14±0.17	0.12±0.15	0.23±0.24	0.24±0.25	0.07±0.09	0.27±0.27	0.07±0.09
6	0.12±0.15	0.07±0.10	0.14±0.17	0.11±0.13	0.25±0.25	0.32±0.30	0.08±0.09	0.26±0.26	0.06±0.09
7	0.12±0.14	0.07±0.10	0.13±0.16	0.11±0.13	0.24±0.24	0.24±0.26	0.07±0.09	0.27±0.27	0.08±0.1
8	0.13±0.15	0.07±0.10	0.13±0.16	0.11±0.13	0.25±0.26	0.25±0.26	0.07±0.09	0.27±0.27	0.06±0.08
9	0.14±0.16	0.07±0.10	0.14±0.18	0.11±0.14	0.23±0.24	0.24±0.25	0.09±0.11	0.27±0.27	0.07±0.09
10	0.13±0.15	0.06±0.09	0.13±0.16	0.11±0.14	0.27±0.26	0.30±0.31	0.07±0.09	0.26±0.25	0.06±0.08
11	0.14±0.16	0.07±0.10	0.14±0.17	0.11±0.14	0.22±0.23	0.27±0.27	0.08± 0.09	0.28±0.26	0.06±0.09
12	0.12±0.14	0.07±10	0.14±0.16	0.12±0.14	0.23±0,24	0.24±0.25	0.08±0.1	0.28±0.27	0,06±0.08
13	0.12±0.14	0.07±0.11	0.13±0.16	0.10±0.12	0.24±0.25	0.30±0.29	0.07±0.09	0.26±0.26	0.06±0.08
14	0.12±0.15	0.07±0.10	0.12±0.15	0.10±0.13	0.24±0.24	0.24±0.25	0.07±0.09	0.26±0.26	0.06±0.09
15	0.12±0.14	0.07±0.10	0.13±0.16	0.11±0.13	0.25±0.25	0.24±0.25	0.08±0.1	0.26±0.26	0.06±0.08
16	0.17±0.18	0.08±0.10	0.21±0.20	0.08±0.10	0.23±0.25	0.28±0.29	0.09±0.1	0.20±0.19	0.08±0.1

17	0.12±0.18	0.07±0.10	0.13±0.17	0.11±0.14	0.26±0.25	0.28±0.29	0.07±0.09	0.28±0.27	0.07±0.09
18	0.13±0.15	0.06±0.09	0.14±0.16	0.11±0.13	0.23±0.24	0.30±0.29	0.08±0.1	0.29±0.28	0.06±0.08
19	0.12±0.15	0.06±0.09	0.12±0.15	0.11±0.14	0.23±0.24	0.25±0.26	0.07±0.09	0.26±0.26	0.06±0.08
20	0.12±0.15	0.07±0.10	0.14±0.17	0.11±0.14	0.25±0.25	0.24±0.25	0.07±0.09	0.26±0.26	0.06±0.09
21	0.12±0.14	0.07±0.11	0.13±0.17	0.11±0.14	0.25±0.25	0.28±0.29	0.07±0.09	0.26±0.26	0.06±0.08
22	0.12±0.15	0.07±0.10	0.12±0.15	0.12±0.14	0.25±0.25	0.25±0.26	0.07±0.09	0.26±0.26	0.07±0.09
23	0.13±0.15	0.08±0.12	0.14±0.18	0.12±0.15	0.23±0.24	0.24±0.25	0.08±0.1	0.27±0.27	0.06±0.08
24	0.12±0.15	0.07±0.11	0.14±0.17	0.12±0.14	0.26±0.27	0.26±0.28	0.08±0.1	0.27±0.27	0.06±0.08
25	0.13±0.16	0.07±0.10	0.14±0.16	0.11±0.15	0.26±0.26	0.29±0.29	0.08±0.1	0.27±0.27	0.07±0.09
26	0.13±0.15	0.07±0.10	0.17±0.20	0.11±0.14	0.21±0.23	0.23±0.24	0.07±0.09	0.26±0.26	0.07±0.09
27	-	0.08±0.12	-	0.12±0.15	0,24±0.25	0.26±0.28	0.08±0.1	0.28±0.28	0.07±0.1
28	-	0.07±0.11	-	0.11±0.13	0.23±0.24	0.24±0.25	0.07±0.09	0.30±0.28	0.06±0.08

Chr	Ga-Matsepe	Dipakakeng	Mgababa	Strydkraal	Modimolle	Ten-Morgan	Magakadimeng
1	0.17±0.19	0.13±0.15	0.06±0.08	0.04±0.05	0.25±0.25	0.13±0.16	0.28±0.28
2	0.17±0.19	0.13±0.16	0.06±0.08	0.04±0.05	0.25±0.25	0.13±0.15	0.28±0.27
3	0.17±0.19	0.14±0.16	0.06±0.08	0.03±0.05	0.25±0.25	0.12±0.15	0.27±0.26
4	0.17±0.19	0.13±0.16	0.06±0.07	0.03±0.05	0.25±0.26	0.12±0.15	0.28±0.27

5	0.17±0.19	0.14±0.17	0.06±0.08	0.04±0.05	0.25±0.26	0.13±0.15	0.26±0.26
6	0.17±0.19	0.12±0.15	0.06±0.08	0.03±0.05	0.28±0.28	0.13±0.15	0.28±0.27
7	0.18±0.21	0.13±0.16	0.06±0.08	0.04±0.05	0.24±0.24	0.13±0.15	0.26±0.27
8	0.20±0.22	0.12±0.15	0.06±0.07	0.03±0.05	0.25±0.25	0.13±0.16	0.27±0.26
9	0.18±0.20	0.14±0.17	0.05±0.07	0.04±0.05	0.26±0.26	0.13±0.16	0.27±0.26
10	0.17±0.19	0.12±0.15	0.06±0.07	0.03±0.05	0.29±0.27	0.13±0.16	0.27±0.27
11	0.16±0.18	0.13±0.16	0.06±0.08	0.03±0.04	0.22±0.23	0.13±0.15	0.27±0.27
12	0.17±0.19	0.15±0.17	0.06±0.08	0.04±0.05	0.23±0.24	0.12±0.15	0.27±0.27
13	0.16±0.18	0.13±0.15	0.06±0.08	0.03±0.05	0.23±0.24	0.13±0.15	0.27±0.27
14	0.17±0.19	0.15±0.17	0.06±0.08	0.03±0.04	0.23±0.24	0.13±0.15	0.28±0.27
15	0.18±0.20	0.15±0.18	0.06±0.08	0.03±0.05	0.25±0.25	0.13±0.15	0.26±0.26
16	0.17±0.16	0.13±0.13	0.06±0.07	0.04±0.06	0.35±0.33	0.20±0.21	0.29±0.29
17	0.16±0.18	0.13±0.16	0.06±0.08	0.03±0.05	0.23±0.24	0.13±0.15	0.27±0.26
18	0.18±0.20	0.13±0.16	0.05±0.07	0.03±0.05	0.26±0.25	0.14±0.16	0.28±0.27
19	0.17±0.19	0.13±0.15	0.06±0.08	0.04±0.05	0.22±0.23	0.13±0.16	0.26±0.26
20	0.17±0.19	0.14±0.16	0.05±0.07	0.03±0.04	0.23±0.24	0.13±0.16	0.27±0.26
21	0.16±0.18	0.12±0.15	0.05±0.07	0.04±0.05	0.27±0.27	0.11±0.14	0.27±0.27

22	0.16±0.18	0.14±0.17	0.05±0.07	0.03±0.05	0.28±0.27	0.14±0.16	0.27±0.27
23	0.17±0.19	0.13±0.16	0.06±0.08	0.03±0.05	0.23±0.24	0.12±0.14	0.27±0.27
24	0.15±0.16	0.15±0.17	0.06±0.08	0.03±0.05	0.25±0.26	0.14±0.16	0.28±0.27
25	0.18±0.20	0.21±0.21	0.06±0.08	0.04±0.05	0.23±0.24	0.13±0.15	0.27±0.27
26	0.17±0.20	0.13±0.16	0.06±0.08	0.04±0.05	0.23±0.24	0.12±0.15	0.26±0.26
27	18±0.20	0.11±0.14	0.06±0.07	0.03±0.04	0.28±0.28	0.12±0.14	0.28±0.27
28	0.16±0.19	0.15±0.17	0.06±0.09	0.03±0.05	0.22±0.24	0.14±0.16	0.27±0.27
