# Supplementary material for: The risk status, signatures of adaptation, and environmental suitability of village-based indigenous chickens from certain regions of Limpopo and KwaZulu-Natal provinces of South Africa
Source: Front Genet. 2024 Dec 18;15:1450939. doi: 10.3389/fgene.2024.1450939 (PMC11688331; doi:10.3389/fgene.2024.1450939)
Supplement: Supplementary file 2 [file Table1.docx]

Supplementary Material

**The risk status, signatures of selection, and environmental suitability of village-based indigenous chickens from certain regions of Limpopo and KwaZulu-Natal provinces of South African**

**Reneilwe Rose Mogano^1, 2*^ Takalani Judas Mpofu^1^ Bohani Mtileni^1 |^ Khanyisile Hadebe^2^**

*** Correspondence:** Khanyisile Hadebe: [MdladlaK@arc.agric.za](mailto:MdladlaK@arc.agric.za)

| **Variables** | **Description** | **Code** |
| --- | --- | --- |
| Soil Variables | Soil pH (×10i n H_2_O) | Soil pH |
|  | Soil sand content (Weight %) | Soil sand content |
|  | Soil silt content (Weight %) | Soil silt content |
|  | Soil clay content (Weight %) | Soil clay content |
|  | Soil water availability capacity (Volume %) | Soil water availability capacity |
|  | Soil bulk density (Kg /m3) | Soil bulk density |
|  | Soil organic carbon (g/kg) | Soil organic carbon |
| Temperature | Annual mean temperature (^0^C) | BIO1 |
|  | Mean Diurnal range(^0^C) | BIO2 |
|  | Isothermility [(BIO2/BIO7)*100)] (^0^C) | BIO3 |
|  | Temperature seasonality (standard deviation^100)( ^0^C) | BIO4 |
|  | Max Temperature of the warmest month (^0^C) | BIO5 |
|  | Min Temperature of the coldest month (^0^C) | BIO6 |
|  | Temperature annual range (BIO5-BIO6)( ^0^C) | BIO7 |
|  | Mean Temperature of wettest quarter (^0^C) | BIO8 |
|  | Mean Temperature of driest quarter (^0^C) | BIO9 |
|  | Mean Temperature of warmest quarter(^0^C) | BIO10 |
|  | Mean Temperature of coldest quarter(^0^C) | BIO11 |
| Solar radiation | Monthly solar radiation (KJ m^-2^ day^-1^) | - |
| Precipitation | Annual precipitation (mm) | BIO12 |
|  | Precipitation of wettest month (mm) | BIO13 |
|  | Precipitation driest month(mm) | BIO14 |
|  | Precipitation of seasonality (mm) | BIO15 |
|  | Precipitation of wettest quarter (mm) | BIO16 |
|  | Precipitation of Driest Quarter (mm) | BIO17 |
|  | Precipitation of Warmest Quarter (mm) | BIO18 |
|  | Precipitation of Coldest Quarter (mm) | BIO19 |

**AAppendix**

Table 2: RDA Identified 368 outliers SNPs related to environmental variables.

| **Chr** | **Outlier SNPs** | **SNP position** | **Axis** | **Gene** | **function** | **Predictor variable** | **correlation** | **Reference** |
| --- | --- | --- | --- | --- | --- | --- | --- | --- |
| 0 | GGaluGA000438_A | 0 | 1 | - |  | Solar radiation of the 7^th^ month | 0.570932775 |  |
| 0 | GGaluGA000689_G | 0 | 1 | - |  | Solar radiation of the 7^th^ month | 0.259755885 |  |
| 0 | GGaluGA001016_G | 0 | 1 | - |  | Soil pH | 0.668014831 |  |
| 0 | GGaluGA001283_A | 0 | 1 | - |  | Solar radiation of the 7^th^ month | 0.298274267 |  |
| 0 | GGaluGA001691_G | 0 | 1 | - |  | Soil pH | 0.612309645 |  |
| 0 | GGaluGA001719_G | 0 | 1 | - |  | Soil pH | 0.577008153 |  |
| 0 | GGaluGA333626_A | 0 | 1 | - |  | Soil pH | 0.668014831 |  |
| 0 | GGaluGA345101_G | 0 | 1 | - |  | Soil cation exchange | 0.339741136 |  |
| 0 | GGaluGA345127_A | 0 | 1 |  |  | Soil pH | 0.668014831 |  |
| 0 | GGaluGA345140_A | 0 | 1 | - |  | Solar radiation of the 7^th^ month | 0.411297083 |  |
| 0 | GGaluGA345167_G | 0 | 1 | - |  | Soil pH | 0.583394715 |  |
| 0 | GGaluGA345246_A | 0 | 1 | - |  | Solar radiation of the 7^th^ month | 0.612114102 |  |
| 0 | GGaluGA345253_A | 0 | 1 | - |  | Solar radiation of the 7^th^ month | 0.556320967 |  |
| 0 | GGaluGA345258_A | 0 | 1 | - |  | Soil pH | 0.603772813 |  |
| 0 | GGaluGA345360_A | 0 | 1 | - |  | Solar radiation of the 7^th^ month | 0.532212232 |  |
| 0 | GGaluGA345366_A | 0 | 1 |  |  | Soil cation exchange | 0.363076928 |  |
| 0 | GGaluGA345372_G | 0 | 1 | - |  | Soil pH | 0.668014831 |  |
| 0 | GGaluGA345380_A | 0 | 1 | - |  | Solar radiation of the 7^th^ month | 0.492100334 |  |
| 0 | GGaluGA345461_A | 0 | 1 | - |  | Soil pH | 0.522567433 |  |
| 0 | GGaluGA345485_G | 0 | 1 | - |  | Soil pH | 0.665484501 |  |
| 0 | GGaluGA345489_G | 0 | 1 | - |  | BIO11 | 0.566896075 |  |
| 0 | GGaluGA345495_C | 0 | 1 | - |  | Solar radiation of the 7^th^ month | 0.574177515 |  |
| 0 | GGaluGA345518_A | 0 | 1 | - |  | Soil cation exchange | 0.398343585 |  |
| 0 | GGaluGA345540_G | 0 | 1 | - |  | Soil organic carbon content | 0.540597403 |  |
| 0 | GGaluGA345588_A | 0 | 1 | - |  | Solar radiation of the 7^th^ month | 0.607095436 |  |
| 0 | GGaluGA345589_G | 0 | 1 | - |  | Solar radiation of the 7^th^ month | 0.609720876 |  |
| 0 | GGaluGA345757_G | 0 | 1 | - |  | Soil pH | 0.532921944 |  |
| 0 | GGaluGA354605_A | 0 | 1 | - |  | Solar radiation of the 7^th^ month | 0.609462436 |  |
| 0 | GGaluGA354675_C | 0 | 1 | - |  | Solar radiation of the 7^th^ month | 0.601339376 |  |
| 0 | GGaluGA360289_A | 0 | 1 | - |  | Solar radiation of the 7^th^ month | 0.61125605 |  |
| 0 | GGaluGA360310_G | 0 | 1 | - |  | Solar radiation of the 7^th^ month | 0.601339376 |  |
| 0 | GGaluGA360405_A | 0 | 1 | - |  | Soil pH | 0.581089594 |  |
| 0 | GGaluGA360443_A | 0 | 1 | - |  | Solar radiation of the 7^th^ month | 0.538015863 |  |
| 0 | GGaluGA360458_A | 0 | 1 | - |  | Solar radiation of 7^th^ month | 0.606385828 |  |
| 0 | GGaluGA360482_A | 0 | 1 | - |  | Solar radiation of the 7^th^ month | 0.53588369 |  |
| 0 | Gga_rs16027111_C | 222183 | 1 | - |  | Soil pH | 0.532921944 |  |
| 0 | GGaluGA344885_G | 448990 | 1 | - |  | Solar radiation of the 7^th^ month | 0.583812358 |  |
| 27 | Gga_rs14302623_A | 2610339 | 1 | *MAPT* | It is located in the nervous system and is involved in the process of microtubule stabilization as well as the formation of the cytoskeleton. | Solar radiation of the 7^th^ month | 0.609462436 | (Cha, Kim and Mook-Jung, 2015; Bateman *et al.*, 2023) |
| 0 | GGaluGA347263_G | 7690473 | 1 | - |  | Soil pH | 0.59855582 |  |
| 1 | Gga_rs13861394_G | 48676526 | 1 | *GRIN2B* | Function as a ligand-gated ion channel in the central nervous system, has a role in excitatory synaptic transmission. | BIO8 | 0.305255438 | (Bateman *et al.*, 2023) |
| 1 | Gga_rs15366328_A | 106836558 | 1 | - |  | Solar radiation of the 7^th^ month | 0.604503473 |  |
| 1 | Gga_rs13924905_G | 117316055 | 1 | *ENSGALG00010003521* |  | Solar radiation of the 7^th^ month | 0.343526703 |  |
| 1 | GGaluGA042731_A | 128521686 | 1 | - |  | Soil cation exchange | 0.460931816 |  |
| 1 | GGaluGA044867_G | 136321669 | 1 | - |  | Solar radiation of the 7^th^ month | 0.301383 |  |
| 1 | GGaluGA044867_G | 136321669 | 1 | *GCC2* | Encodes a membrane protein localized to the trans-Golgi network and is related to the vesicular transport between the endosomes and the Golgi. | Solar radiation of the 7^th^ month | 0.301382609 | ( Wu *et al.*, 2022) |
| 1 | Gga_rs13947014_A | 142307262 | 1 | *ENSGALG00010006221* |  | Solar radiation of the 7^th^ month | 0.466738322 |  |
| 1 | Gga_rs15462937_A | 154513024 | 1 | - |  | BIO8 | 0.376918616 |  |
| 1 | Gga_rs15523624_C | 183681253 | 1 | *CNTN5* | Mediate cell surface interactions during nervous system development. | Soil pH | 0.490807038 | (Bateman *et al.*, 2023) |
| 2 | GGaluGA167394_A | 130300569 | 1 | - |  | BIO8 | 0.305351506 |  |
| 2 | GGaluGA170562_A | 142400711 | 1 | - |  | Soil cation exchange | 0.390399739 |  |
| 3 | GGaluGA206756_G | 9514328 | 1 | *EHBP1* | Required for perinuclear sorting and insulin-regulated recycling of SLC2A4/GLUT4 in adipocytes. | BIO8 | 0.326132522 | (Guilherme *et al.*, 2004) |
| 3 | GGaluGA216016_A | 35506417 | 1 | *OPN3* | Is associated with light-independent functions such as the regulation of melanogenesis and apoptosis in epidermal melanocytes, may be involved in photo relaxation of airway smooth muscle cells. | Solar radiation of the 7^th^ month | 0.315956633 | (Ozdeslik *et al.*, 2019) |
| 3 | Gga_rs14347250_G | 45039910 | 1 | *-* |  | BIO8 | 0.38854121 |  |
| 3 | Gga_rs14356816_C | 50829540 | 1 | - |  | BIO11 | 0.430724931 |  |
| 3 | Gga_rs15359681_A | 55542623 | 1 | *ENSGALG00010006629* |  | Soil cation exchange | 0.332269176 |  |
| 3 | GGaluGA229903_G | 79969785 | 1 | *ENSGALG00010004882* |  | Soil pH | 0.300978597 |  |
| 4 | Gga_rs15597184_G | 62785313 | 1 | *FGF20* | A novel central player in the development of vertebrate skin appendages, including hair follicles and exocrine glands. | Solar radiation of the 7^th^ month | 0.347410908 | (Wells *et al.*, 2012; Biggs *et al.*, 2018) |
| 4 | GGaluGA268598_A | 86602861 | 1 | - |  | Soil pH | 0.663042228 |  |
| 5 | Gga_rs16468580_G | 14556016 | 1 | *MUC2* | Responsible for lubrication and protection of the external surface of the internal epithelium tissue. | BIO11 | 0.515664 | (Bateman *et al.*, 2023) |
| 5 | GGaluGA283462_A | 39569745 | 1 | *NRXN3* | Is involved in cell recognition and cell adhesion, plays a role in angiogenesis | Solar radiation of the 7^th^ month | 0.351916 | (Bottos *et al.*, 2009) |
| 5 | GGaluGA284084_A | 40771771 | 1 | - |  | Soil pH | 0.568704 |  |
| 5 | Gga_rs15736049_A | 56204029 | 1 | *ENSGALG00010020239* |  | Solar radiation of the 7^th^ month | 0.394916 |  |
| 5 | Gga_rs14552339_G | 57143305 | 1 | *ENSGALG00010020247* |  | Soil cation exchange | 0.338525 |  |
| 6 | GGaluGA304457_A | 29878437 | 1 | *ENSGALG00010023121* |  | BIO8 | 0.456257 |  |
| 6 | GGaluGA305836_A | 33039706 | 1 | *CTBP2* | Function in brown adipose tissue (BAT) differentiation. | BIO8 | 0.347642 | (Bateman *et al.*, 2023) |
| 8 | Gga_rs15911039_A | 13112398 | 1 | *TLCD4* | Is involved in lipid trafficking and metabolism. | Solar radiation of the 7^th^ month | 0.402395 | (Attwood and Schiöth, 2021) |
| 8 | Gga_rs14652414_G | 24095217 | 1 | *TUT4* | Essential for both oocyte maturation and fertility. | Soil pH | 0.348098 | (Bateman *et al.*, 2023) |
| 9 | Gga_rs14663375_C | 10399040 | 1 | *SLC9A9* | Is responsible for insulin-induced glucose uptake by muscle and adipose tissue. | Solar radiation of the 7^th^ month | 0.431287 | (Zhuang *et al.*, 2022) |
| 9 | GGaluGA344028_G | 24218624 | 1 | - |  | BIO8 | 0.344037 |  |
| 10 | Gga_rs15559396_C | 1153367 | 1 | - |  | BIO11 | 0.464416 |  |
| 10 | GGaluGA070801_G | 15680150 | 1 | *ND2* | Participates in mitochondrial respiratory chain and oxidative phosphorylation, involved in energy metabolism. | Soil cation exchange | 0.483112 | (Barker *et al.*, 2012; Yang *et al.*, 2020) |
| 11 | Gga_rs14022503_G | 9324097 | 1 | *ENSGALG00010025540* |  | Soil pH | 0.45396 |  |
| 11 | Gga_rs14964520_A | 12002435 | 1 | *ENSGALG00010010192* |  | Solar radiation of 7^th^ month | 0.601339 |  |
| 12 | Gga_rs14692320_A | 14147 | 1 | - |  | Solar radiation of the 7^th^ month | 0.611979 |  |
| 12 | Gga_rs14029638_G | 35026 | 1 | *ENSGAG00010028209* |  | BIO11 | 0.486083 |  |
| 12 | GGaluGA080925_G | 1040195 | 1 | - |  | Solar radiation of 7^th^ month | 0.39556 |  |
| 12 | Gga_rs15656424_A | 12454240 | 1 | *FHIT* | Is associated with body mass index, and may be associated with carcinogenesis. | BIO8 | 0.339138 | (Joannes *et al.*, 2014; Ahmad *et al.*, 2016) |
| 12 | GGaluGA087997_G | 16378623 | 1 | *FOXP1* | Regulates gene transcription, controls adipocyte differentiation, and plays a tissue-specific role. | Solar radiation of 7^th^ month | 0.357006 | (Liu *et al.*, 2019; Bateman *et al.*, 2023) |
| 13 | Gga_rs14060730_A | 12317148 | 1 | - |  | Solar radiation of the 7^th^ month | 0.485896 |  |
| 13 | GGaluGA096104_A | 13280239 | 1 | *ADAMTS2* | It process the procollagens I, II, and III's amino-propeptides; and may be necessary for the normal deposition of collagen fibrils. | Soil cation exchange | 0.46034 | (Bekhouche and Colige, 2015) |
| 13 | Gga_rs29007783_G | 18860595 | 1 | - |  | Solar radiation of 7^th^ month | 0.59197 |  |
| 14 | Gga_rs15003166_G | 1082003 | 1 | *LMTK2* | Involved in neuronal outgrowth and development, axonal transport, intracellular vesicle trafficking, and apoptosis. | Soil pH | 0.401391 | (Chibalina *et al.*, 2007; Manser *et al.*, 2012; Conti *et al.*, 2017) |
| 14 | GGaluGA104361_A | 12967732 | 1 | - |  | Solar radiation of the 7^th^ month | 0.348991 |  |
| 15 | GGaluGA106699_A | 2030299 | 1 | - |  | Soil pH | 0.275557 |  |
| 15 | Gga_rs15020396_G | 5302531 | 1 |  |  | Soil pH | 0.322798 |  |
| 17 | GGaluGA114611_G | 5399508 | 1 | - |  | Soil cation exchange | 0.417505 |  |
| 18 | GGaluGA119634_A | 3815304 | 1 | *B3GNTL1* | Is a putative glycosyltransferase. | Solar radiation of the 7^th^ month | 0.32445 | (Wu *et al.*, 2022) |
| 20 | Gga_rs15170561_G | 2747608 | 1 | *TOX2* | Putative transcriptional activator involved in the hypothalamus-pituitary-gonadal system. | Solar radiation of the 7^th^ month | 0.287132 | (Bateman *et al.*, 2023) |
| 20 | Gga_rs16172230_A | 10301225 | 1 | - |  | Soil cation exchange | 0.311473 |  |
| 20 | GGaluGA180443_A | 10438342 | 1 | *XKR7* | The biochemical function and tissue distribution of members of the Xkr family are not well understood. | Solar radiation of the 7^th^ month | 0.498692 | (Suzuki, Imanishi and Nagata, 2014) |
| 23 | Gga_rs14291225_G | 4804301 | 1 | - |  | Solar radiation of the 7^th^ month | 0.601339 |  |
| 23 | Gga_rs13623028_A | 6005041 | 1 | *MYOM3* | Involved in binding of myosin to other proteins such as titin, stabilizing the thick filament lattice and the sarcomere structure (found in striated muscles). | Solar radiation of 7^th^ month | 0.336885 | (Schoenauer *et al.*, 2008) |
| 26 | Gga_rs16346673_A | 8011 | 1 | - |  | Soil pH | 0.668015 |  |
| 26 | GGaluGA196819_G | 2987508 | 1 | *PLXNA2* | Play a role in axon guidance invasive growth and cell migration | Solar radiation of the 7^th^ month | 0.357965 | (Bateman *et al.*, 2023) |
|  | **RDA2** |  |  |  |  |  |  |  |
| 0 | GGaluGA000051_G | 0 | 2 | - |  | BIO11 | 0.358782 |  |
| 0 | GGaluGA000319_A | 0 | 2 | - |  | BIO11 | 0.302977 |  |
| 0 | GGaluGA000580_A | 0 | 2 | - |  | BIO11 | 0.268772 |  |
| 0 | GGaluGA000840_A | 0 | 2 | - |  | BIO11 | 0.190498 |  |
| 0 | GGaluGA000872_A | 0 | 2 | - |  | BIO6 | 0.409833 |  |
| 0 | GGaluGA001396_G | 0 | 2 | - |  | BIO11 | 0.342572 |  |
| 0 | GGaluGA001797_G | 0 | 2 | - |  | Soil organic carbon | 0.608252 |  |
| 0 | GGaluGA271401_A | 0 | 2 | - |  | Soil organic carbon | 0.644419 |  |
| 0 | GGaluGA345283_A | 0 | 2 | - |  | BIO6 | 0.431741 |  |
| 0 | GGaluGA345365_A | 0 | 2 | - |  | BIO11 | 0.275914 |  |
| 0 | GGaluGA345416_A | 0 | 2 | - |  | Soil organic carbon | 0.644419 |  |
| 0 | GGaluGA360195_A | 0 | 2 | - |  | BIO11 | 0.383779 |  |
| 0 | GGaluGA360247_G | 0 | 2 | - |  | BIO11 | 0.319337 |  |
| 0 | GGaluGA360297_G | 0 | 2 | - |  | Soil organic carbon | 0.598915 |  |
| 0 | GGaluGA360452_A | 0 | 2 |  |  | Soil organic carbon | 0.425539 |  |
| 0 | Gga_rs14725137_G | 292354 | 2 | - |  | BIO6 | 0.264881 |  |
| 0 | Gga_rs14689052_A | 389095 | 2 | - |  | BIO11 | 0.284844 |  |
| 0 | Gga_rs13772846_G | 579201 | 2 | - |  | BIO6 | 0.375454 |  |
| 28 | Gga_rs16209716_G | 684728 | 2 | - |  | Elevation | 0.297659 |  |
| 28 | GGaluGA201504_G | 1474215 | 2 | *MFSD12* | It regulates skin and hair pigmentation. | BIO11 | 0.329363 | (Hédan *et al.*, 2019) |
| 27 | Gga_rs16719252_C | 2956025 | 2 | - |  | Soil clay content | 0.141902 |  |
| 27 | Gga_rs14302011_A | 2012987 | 2 | *TLK2* | Involved in DNA replication, DNA repair, transcription, chromatin structure, chromosome segregation, RNA interference, viral latency, and cell cycle checkpoint. | Elevation | 0.238691 | (Bateman *et al.*, 2023) |
| 27 | GGaluGA200100_G | 3731373 | 2 | - |  | BIO6 | 0.352326 |  |
| 27 | GGaluGA200497_A | 4458047 | 2 | - |  | Soil organic carbon | 0.481089 |  |
| 0 | Gga_rs16765066_G | 26304442 | 2 | - |  | BIO6 | 0.472365 |  |
| 0 | Gga_rs16123132_A | 27929968 | 2 | - |  | BIO11 | 0.542869 |  |
| 0 | Gga_rs14778854_C |  | 2 | - |  | BIO11 | 0.605303 |  |
| 1 | GGaluGA003981_G | 6583362 | 2 | - |  | BIO6 | 0.369874 |  |
| 1 | Gga_rs15200439_G | 13158546 | 2 | - |  | BIO6 | 0.34756 |  |
| 1 | Gga_rs13842165_A | 28800862 | 2 | *NRCAM* | Involved in the development of optic fibres in the retina. | BIO11 | 0.251643 | (Bateman *et al.*, 2023) |
| 1 | Gga_rs14803801_G | 29831809 | 2 | *PRICKLE1* | Involved in the planar cell polarity pathway. | BIO6 | 0.384737 | (McNeill and Woodgett, 2010) |
| 1 | GGaluGA010542_G | 31864765 | 2 | *LRIG3* | Plays a role in embryo development, including cranio-facial morphogenesis and neural crest formation. | BIO11 | 0.210361 | (Abraira *et al.*, 2008) |
| 1 | Gga_rs13849591_G | 36348081 | 2 | *LRIG3* | Plays a role in embryo development, including cranio-facial morphogenesis and neural crest formation. | BIO11 | 0.483575 | (Abraira *et al.*, 2008) |
| 1 | Gga_rs13853671_A | 40142197 | 2 | *ENSGALG00010014881* | - | BIO6 | 0.364983 |  |
| 1 | Gga_rs13855683_G | 41875670 | 2 | *ENSGALG00010011199* | - | Soil clay content | 0.158313 |  |
| 1 | GGaluGA023549_G | 69261786 | 2 | *PARVG* | Plays a role in the regulation of cell adhesion and cytoskeleton organization. | BIO6 | 0.241006 | (Bateman *et al.*, 2023) |
| 1 | Gga_rs13883961_G | 71754673 | 2 | *LRP6* | Involved in numerous biological activities such as cell proliferation, specification, migration, metastatic cancer, and embryonic development. | Soil clay content | 0.243693 | (MacDonald and He, 2012) |
| 1 | GGaluGA026119_A | 79592879 | 2 | *STXBP5L* | Plays a role in vesicle trafficking and exocytosis inhibition, neurotransmitter release by inhibiting basal. | Soil clay content | 0.246296 | (Bateman *et al.*, 2023) |
| 1 | Gga_rs13887360_G | 80600766 | 2 | *CHD1L* | Promote cell proliferation, enhance cell migration, and inhibit apoptosis by regulating various complex networks. | BIO6 | 0.335955 | (Xu *et al.*, 2016) |
| 1 | Gga_rs15332915_A | 88873985 | 2 | *C1H3ORF52* | Involved in membrane structure. | BIO6 | 0.346587 | (Cheng *et al.*, 2019) |
| 1 | GGaluGA032419_A | 95433715 | 2 | *GBE1* | Required for normal glycogen accumulation. | BIO11 | 0.258916 | (Chen *et al.*, 2022; Bateman *et al.*, 2023) |
| 1 | Gga_rs13924926_G | 117411374 | 2 | - |  | BIO6 | 0.369914 |  |
| 1 | Gga_rs13924967_G | 117487641 | 2 |  |  | BIO11 | 0.422062 |  |
| 1 | GGaluGA041232_A | 124377338 | 2 | *ARHGAP6* | Regulate actin polymerization during many cellular activities, including tumour cellular. | BIO11 | 0.226669 | (Tribioli *et al.*, 1996; Schaefer *et al.*, 1997) |
| 1 | Gga_rs13937342_A | 132997809 | 2 | *AFF3* | Mediating transcriptional activity, revealing an association with the positive transcription elongation factor b (P-TEFb). | BIO6 | 0.350997 | (Bitoun, Oliver and Davies, 2007) |
| 1 | Gga_rs13940030_G | 135573467 | 2 | *UXS1* | Is unique among the enzymes involved in nucleotide sugar biosynthesis in that it is found within the secretory pathway lumen. | BIO6 | 0.442417 | (Xiao *et al.*, 2020) |
| 1 | Gga_rs13942782_G | 138216261 | 2 | *MYO16* | Serve in intracellular movements, involved in targeting of the catalytic subunit of protein phosphatase 1 during brain development. | BIO11 | 0.314843 | (Bateman *et al.*, 2023) |
| 1 | Gga_rs13712468_C | 149395062 | 2 | - |  | BIO11 | 0.22356 |  |
| 1 | GGaluGA050403_A | 154425848 | 2 | *FBXL3* | Role in the maintenance of both the speed and the robustness of the circadian clock oscillation. | BIO11 | 0.463569 | (Bateman *et al.*, 2023) |
| 1 | GGaluGA051950_G | 160862971 | 2 | - |  | BIO6 | 0.287257 |  |
| 1 | Gga_rs15497877_A | 173593810 | 2 | - |  | BIO11 | 0.337634 |  |
| 1 | GGaluGA057568_A | 180632601 | 2 | - |  | Soil clay content | 0.304238 |  |
| 1 | Gga_rs13981373_G | 181735314 | 2 | - |  | BIO6 | 0.3463 |  |
| 1 | Gga_rs13992644_G | 192077117 | 2 | [*TENM4*](http://www.ensembl.org/gallus_gallus/Gene/Summary?db=core;g=ENSGALG00010009135) | Involved in neural development, regulating the establishment of proper connectivity within the nervous system. | BIO11 | 0.350606 | (Hong, Mosca and Luo, 2012) |
| 1 | GGaluGA062629_G | 195409234 | 2 | - |  | BIO11 | 0.29851 |  |
| 1 | GGaluGA140145_A | 32267173 | 2 | - |  | BIO11 | 0.37032 |  |
| 2 | Gga_rs10729700_A | 32929834 | 2 | [*JAZF1*](http://www.ensembl.org/gallus_gallus/Gene/Summary?db=core;g=ENSGALG00010001458) | Regulates cell growth and proliferation through different physiological pathways, thereby acting as a transcriptional growth factor. | BIO6 | 0.344331 | (Abuderman *et al.*, 2020) |
| 2 | Gga_rs15084136_A | 36031561 | 2 | - |  | BIO11 | 0.213304 |  |
|  | GGaluGA141341_A | 36593043 | 2 | - |  | BIO11 | 0.304566 |  |
| 2 | GGaluGA142667_A | 40162929 | 2 | [*OSBPL10*](http://www.ensembl.org/gallus_gallus/Gene/Summary?db=core;g=ENSGALG00010002334) | Involved in lipid counter transport between the endoplasmic reticulum and the plasma membrane. | BIO11 | 0.294823 | (Bateman *et al.*, 2023) |
| 2 | Gga_rs15088767_A | 41004774 | 2 | - |  | BIO11 | 0.348476 |  |
| 2 | Gga_rs14188183_G | 59222173 | 2 | - |  | BIO6 | 0.392187 |  |
| 2 | Gga_rs14189950_G | 60439301 | 2 | - |  | BIO11 | 0.34779 |  |
| 2 | Gga_rs14190499_G | 60987066 | 2 | [*JARID2*](http://www.ensembl.org/gallus_gallus/Gene/Summary?db=core;g=ENSGALG00010009678) | It is crucial to embryonic development, including heart and liver development, neural tube fusion, and haematopoiesis. | BIO6 | 0.475138 | (Bateman *et al.*, 2023) |
| 2 | Gga_rs14195082_A | 64526132 | 2 | - |  | Soil_pH | 0.202072 |  |
| 2 | Gga_rs15118483_G | 79104255 | 2 | - |  | BIO6 | 0.386874 |  |
| 2 | Gga_rs14220432_A | 94753701 | 2 | - |  | BIO11 | 0.287786 |  |
| 2 | Gga_rs13640047_C | 98545603 | 2 | - |  | BIO6 | 0.34181 |  |
| 2 | GGaluGA159003_G | 98620682 | 2 | [*PTPRM*](http://www.ensembl.org/gallus_gallus/Gene/Summary?db=core;g=ENSGALG00010001940) | Plays a role in adipogenic differentiation. | BIO11 | 0.259757 | (Bateman *et al.*, 2023) |
| 2 | Gga_rs14225325_A | 103152561 | 2 | - |  | BIO11 | 0.382529 |  |
| 2 | GGaluGA161488_A | 106631880 | 2 | - |  | Soil clay content | 0.282233 |  |
| 2 | Gga_rs14232790_A | 112617669 | 2 | *CHD7* | Probable transcription regulator, involved in the 45S precursor rRNA production. | BIO11 | 0.36609 | (Bateman *et al.*, 2023) |
| 2 | Gga_rs14238834_A | 119948911 | 2 | *PKIA* | Interacts with the catalytic subunit of the enzyme after the cAMP-induced dissociation of its regulatory chains. | BIO11 | 0.28614 | (Bateman *et al.*, 2023) |
| 2 | Gga_rs14241422_C | 123037752 | 2 | *-* |  | BIO6 | 0.330965 |  |
| 2 | Gga_rs14249696_G | 131239733 | 2 | *EMC2* | Associated with embryonic development. | BIO11 | 0.336711 | (Fang *et al.*, 2022) |
| 2 | Gga_rs14251874_A | 136204598 | 2 | *-* |  | BIO11 | 0.263556 |  |
| 2 | Gga_rs15158544_G | 139849672 | 2 | *-* |  | BIO6 | 0.401384 |  |
| 2 | GGaluGA170215_G | 141447770 | 2 | *-* |  | BIO6 | 0.386766 |  |
| 2 | GGaluGA170471_G | 142116401 | 2 | *KHDRBS3* | Found to be associated with diaphyseal total bone content, as well as the cortical traits. | BIO11 | 0.539117 | (Johnsson *et al.*, 2015) |
| 2 | GGaluGA171676_C | 145445023 | 2 | *-* |  | BIO6 | 0.26 |  |
| 3 | Gga_rs14315833_G | 8645980 | 2 | *ALK* | Associated with parasite resistance traits. | BIO11 | 0.234763 | (Arzik *et al.*, 2022) |
| 3 | Gga_rs13503629_A | 9528980 | 2 | *EHBP1* | Required for perinuclear sorting and insulin-regulated recycling of SLC2A4/GLUT4 in adipocytes. | BIO6 | 0.262727 | (Guilherme *et al.*, 2004) |
| 3 | Gga_rs13733710_A |  | 2 | *PLCB4* | Play a role in Immune response and heat stress. | Bio6 | 0.393056 | (Jin *et al.*, 2017; Li *et al.*, 2020) |
| 3 | Gga_rs16729741_C |  | 2 | - |  | BIO11 | 0.492655 |  |
| 3 | Gga_rs13723135_A | 34716803 | 2 | *AKT3* | Function in controlling fat and cholesterol composition contributes to immunity processes. | BIO11 | 0.219451 | (Tsiperson *et al.*, 2013; Farmanullah *et al.*, 2020) |
| 3 | GGaluGA216266_G | 36175337 | 2 | *CHRM3* | Mediates cellular responses, inhibiting adenylate cyclase, breaking phosphoinositide’s, and modulating potassium channels through G proteins, with Pi turnover being the primary transducing effect. | BIO6 | 0.382008 | (Bateman *et al.*, 2023) |
| 3 | Gga_rs13724003_A | 36675339 | 2 | *-* |  | BIO11 | 0.339663 |  |
| 3 | Gga_rs14341210_G | 37571945 | 2 | *-* |  | BIO11 | 0.541957 |  |
| 3 | Gga_rs14343948_C | 42212061 | 2 | *RNASET2* | Regulates antioxidant tone and is required for physiological response to reactive oxygen. | Elevation | 0.278347 | (Gondret *et al.*, 2017) |
| 3 | GGaluGA218188_G | 42342712 | 2 | *RPS6KA2* | Is associated with reproduction. | BIO6 | 0.314453 | (Sun *et al.*, 2023) |
| 3 | Gga_rs14367309_A | 65331622 | 2 | - |  | BIO11 | 0.295454 |  |
| 3 | Gga_rs14373013_A | 70766828 | 2 | - |  | BIO11 | 0.336752 |  |
| 3 | Gga_rs14379070_G | 78522143 | 2 | - |  | BIO11 | 0.34352 |  |
| 3 | Gga_rs14389831_G | 89439162 | 2 | *CSMD1* | Acts as a tumour suppressor. | BIO11 | 0.30332 | (Kamal *et al.*, 2017) |
| 3 | Gga_rs14405234_A | 104442900 | 2 | *-* |  | BIO11 | 0.172121 |  |
| 3 | GGaluGA236963_G | 104509695 | 2 | *-* |  | BIO11 | 0.330605 |  |
| 3 | GGaluGA237309_A | 105844475 | 2 | *-* |  | Soil silt content | 0.188737 |  |
| 3 | GGaluGA237957_G | 107670367 | 2 | *-* |  | BIO11 | 0.348504 |  |
| 3 | Gga_rs16348764_G | 1285816 | 2 | *-* |  | BIO6 | 0.287543 |  |
| 4 | Gga_rs15476289_C | 3020921 | 2 | *-* |  | BIO11 | 0.316492 |  |
| 4 | GGaluGA242707_C | 3161005 | 2 | *KLHL9* | Involved in distal myopathy. | BIO6 | 0.308012 | (Cirak *et al.*, 2010) |
| 4 | Gga_rs14430470_A | 12983537 | 2 | - |  | BIO6 | 0.361048 |  |
| 4 | Gga_rs14435465_G | 17485479 | 2 | - |  | Soil clay content | 0.216688 |  |
| 4 | Gga_rs13576865_A | 25230815 | 2 | - |  | BIO6 | 0.421547 |  |
| 4 | Gga_rs14445405_G | 31886929 | 2 | *NR3C2* | Showing features for DNA binding. | BIO6 | 0.350457 | (Bateman *et al.*, 2023) |
| 4 | GGaluGA251938_G | 32448004 | 2 | *LRBA* | Coordinates immune receptor signalling to increase effector function and so plays an important role in immune regulation. | BIO11 | 0.501649 | (Wang *et al.*, 2001) |
| 4 | GGaluGA254118_A | 38194284 | 2 | *GSTCD* | Is associated with lung functionality and respiratory system. | BIO6 | 0.238441 | (Liu *et al.*, 2017; Aboulnaga *et al.*, 2021) |
| 4 | Gga_rs14455367_G | 40713404 | 2 | *-* |  | BIO11 | 0.510365 |  |
| 4 | Gga_rs14462628_A | 47754794 | 2 | *ADGRL3* | Involvement in the brain and the neuronal development and processes. | Elevation | 0.400325 | (Gheyas *et al.*, 2021) |
| 4 | GGaluGA261434_G | 59995702 | 2 | *PPP3CA* | Involved in the calcineurin signalling pathway, which initiates skeletal muscle differentiation. | BIO6 | 0.352845 | (Friday *et al.*, 2003) |
| 4 | Gga_rs14477470_A | 60513755 | 2 | *CENPE* | Plays an important role in chromosome congress ion, microtubule-kinetochore conjugation and spindle assembly checkpoint activation. | BIO11 | 0.289628 | (Bateman *et al.*, 2023) |
| 4 | Gga_rs15601457_G | 65227298 | 2 | *LNX1* | Ubiquitin ligases that interact with Numb a key regulator of neurogenesis and neuronal differentiation. | BIO11 | 0.322165 | (Lenihan *et al.*, 2014) |
| 4 | Gga_rs13550100_G | 68601761 | 2 | *PDS5A* | Regulates muscle cell proliferation and muscle fiber transformation. | BIO11 | 0.255703 | (Wang *et al.*, 2020) |
| 4 | GGaluGA264376_A | 70308348 | 2 | - |  | Soil clay content | 0.218179 |  |
| 4 | Gga_rs16448899_A | 89076844 | 2 | - |  | Elevation | 0.520781 |  |
| 5 | Gga_rs16736715_G | 239332 | 2 | *TMEM132A* | Has a role in both embryonic and postnatal brain development Increased resistance to cell death caused by serum deprivation in cultured cells. | BIO11 | 0.283864 | (Bateman *et al.*, 2023) |
| 5 | GGaluGA272203_G | 1553354 | 2 | - |  | BIO11 | 0.303566 |  |
| 5 | Gga_rs14352346_G | 4989663 | 2 | - |  | Soil clay content | 0.206971 |  |
| 5 | GGaluGA274762_G | 11330406 | 2 | - |  | BIO11 | 0.252395 |  |
| 5 | GGaluGA274797_G | 11421851 | 2 | *PIK3C2A* | Involved with insulin secretion, which is regulated by the insulin receptor and it also participates in the exocytosis of insulin granules. | BIO11 | 0.381737 | (Marchesi *et al.*, 2021) |
| 5 | GGaluGA274944_A | 12139609 | 2 | *GTF2H1* | Involved in DNA repair factor IIH (TFIIH) core complex. | BIO8 | 0.177678 | (Bateman *et al.*, 2023) |
| 5 | GGaluGA275823_C | 14612505 | 2 | - |  | Elevation | 0.285755 |  |
| 5 | GGaluGA280970_G | 29805529 | 2 | *RYR3* | Play a role in triggering muscle contraction. | BIO11 | 0.340215 | (Sporer *et al.*, 2012; Bateman *et al.*, 2023) |
| 5 | GGaluGA282400_G | 35263968 | 2 | - |  | BIO11 | 0.364315 |  |
| 5 | Gga_rs14547172_G | 52581455 | 2 | *SYNE2* | Involved in cardiomyopathy networks. | BIO11 | 0.387993 | (Kharrati-Koopaee *et al.*, 2021) |
| 5 | Gga_rs13590914_A | 52629954 | 2 | [*SYNE2*](http://www.ensembl.org/gallus_gallus/Gene/Summary?db=core;g=ENSGALG00010017621) | Involved in cardiomyopathy networks. | BIO11 | 0.337106 | (Kharrati-Koopaee *et al.*, 2021) |
| 5 | Gga_rs14547414_C | 52775726 | 2 | *WDR89* | It is essential for the elongation step of protein synthesis. | BIO11 | 0.486733 | (Chen *et al.*, 2021) |
| 6 | GGaluGA294542_G | 4710384 | 2 | *ENSGALG00010011328* |  | Elevation | 0.669031 |  |
| 6 | Gga_rs16537473_G | 8712763 | 2 | *ENSGALG00010011380* |  | BIO11 | 0.45859 |  |
| 6 | Gga_rs14578424_A | 17983333 | 2 | *ENSGALG00010023741* |  | BIO6 | 0.433617 |  |
| 6 | Gga_rs10727195_A | 27267941 | 2 | *ENSGALG00010023085* |  | BIO6 | 0.445292 |  |
| 6 | Gga_rs16558943_A | 28062984 | 2 | *ENSGALG00010023092* |  | BIO11 | 0.323245 |  |
| 6 | Gga_rs14590924_G | 31003444 | 2 | *RGS10* | Function as GTPase activating protein (GAP) for Gαi family of G-proteins, highly expressed in the central nervous system (CNS) and immune system. | BIO11 | 0.376108 | (Waugh *et al.*, 2005) |
| 7 | Gga_rs16571933_G | 1609228 | 2 | *ENSGALG00010015519* |  | BIO11 | 0.435926 |  |
| 7 | Gga_rs14600805_G | 3229998 | 2 | *ERBB4* | Involved in the calcium signalling pathway, regulators of neural and muscle development, and also involved in fear response. | BIO11 | 0.447538 | (Chen *et al.*, 2017; Ren *et al.*, 2018) |
| 7 | Gga_rs14608371_A | 13219459 | 2 | *ABI2* | Is associated with muscle development. | Soil Organic carbon | 0.201431 | (Ma *et al.*, 2022) |
| 7 | GGaluGA312831_A | 14595204 | 2 | - |  | BIO11 | 0.233066 |  |
| 7 | Gga_rs14610948_G | 17223472 | 2 | - |  | BIO11 | 0.530392 |  |
| 7 | Gga_rs14613256_A | 20726831 | 2 | *FAP* | Associated with proliferation and function of fibroblasts. | BIO8 | 0.194261 | (Papah *et al.*, 2018) |
| 7 | Gga_rs16594933_A | 22446374 | 2 | - |  | BIO11 | 0.308584 |  |
| 7 | GGaluGA316024_G | 24505528 | 2 | *ENSGALG00010022080* |  | BIO11 | 0.32516 |  |
| 7 | GGaluGA318577_A | 30047727 | 2 | [*ACMSD*](http://www.ensembl.org/gallus_gallus/Gene/Summary?db=core;g=ENSGALG00010019550) | Encodes for a critical enzyme of the kynurenine pathway of the tryptophan metabolism. | BIO11 | 0.293359 | (Martí-Massó *et al.*, 2013; Bateman *et al.*, 2023) |
| 7 | GGaluGA320978_C | 36515245 | 2 | - |  | BIO11 | 0.313398 |  |
| 7 | GGaluGA321196_A | 36958722 | 2 | - |  | Soil clay content | 0.314136 |  |
| 7 | GGaluGA321212_A | 36979435 | 2 | - |  | Soil Organic carbon | 0.169888 |  |
| 8 | Gga_rs14633891_C | 2430283 | 2 | *DENND1B* | It regulate cell inflammation and apoptosis by sponging miR-30e-3p. | BIO11 | 0.528472 | (Chen *et al.*, 2021) |
| 8 | GGaluGA322786_A | 3591953 | 2 | *RGS4* | Is associated with myometrium relaxation. | BIO11 | 0.294681 | (Zwane *et al.*, 2021) |
| 8 | GGaluGA324393_G | 6475929 | 2 | *RASAL2* | Metabolic regulator involved in energy homeostasis and adipogenesis. | BIO11 | 0.291841 | (Ding *et al.*, 2021) |
| 8 | GGaluGA325049_G | 7385552 | 2 | - |  | BIO11 | 0.238475 |  |
| 8 | GGaluGA325424_G | 8018804 | 2 | *FAM129A* | Is found to be associated with abdominal fat weight. | BIO11 | 0.322506 | (Zhang *et al.*, 2020) |
| 8 | Gga_rs16632921_G | 16860915 | 2 | *ENSGALG00010024405* |  | BIO11 | 0.41579 |  |
| 8 | Gga_rs14645777_C | 17356752 | 2 | *ENSGALG00010024443* |  | BIO11 | 0.192217 |  |
| 9 | Gga_rs14669338_A | 5293361 | 2 | *PIK3CB* | Is associated with white/red earlobe colour formation. | BIO6 | 0.217912 | (Luo *et al.*, 2018) |
| 9 | Gga_rs14667573_A | 6842640 | 2 | *ENSGALG00010026146* |  | Elevation | 0.276946 |  |
| 9 | GGaluGA336941_A | 7380996 | 2 | *EPHA4* | Plays a role in the development of the nervous system controlling different steps of axonal guidance. | BIO11 | 0.435702 | (Bateman *et al.*, 2023) |
| 9 | Gga_rs13765098_G | 9064184 | 2 | *COL4A3* | Play an important role in the structural maintenance and normal development of skeletal muscle. | BIO11 | 0.394519 | (Baumert *et al.*, 2018; Mohammadkhah, Murphy and Simms, 2018) |
| 9 | GGaluGA338995_G | 12812899 | 2 | *ATP13A5* | Associated with biological processes important for lipid metabolism and the development of skeletal muscle. | BIO6 | 0.273273 | (Zhang *et al.*, 2017) |
| 9 | Gga_rs13736133_G | 22577221 | 2 | *TIPARP* | Is associated with a favourable prognosis. | Soil clay content | 0.239674 | (Zhang *et al.*, 2020) |
| 10 | GGaluGA065293_A | 543396 | 2 | - |  | BIO11 | 0.427638 |  |
| 10 | GGaluGA073231_A | 21780573 | 2 | - |  | BIO11 | 0.343209 |  |
| 10 | GGaluGA073413_A | 22058298 | 2 | - |  | BIO6 | 0.356646 |  |
| 11 | Gga_rs14020878_A | 6827222 | 2 | - |  | BIO11 | 0.414842 |  |
| 11 | Gga_rs14021051_A | 7330533 | 2 | *TENM2* | Involved in neural development, regulating the establishment of proper connectivity within the nervous system. | BIO6 | 0.365335 | (Fleming *et al.*, 2016) |
| 11 | Gga_rs14028860_A | 20340084 | 2 | - |  | BIO6 | 0.234665 |  |
| 12 | GGaluGA083711_G | 6897734 | 2 | *FGD3* | Is associated with birth weight. | BIO11 | 0.330315 | (Takasuga *et al.*, 2015; Stronen *et al.*, 2019) |
| 12 | GGaluGA087987_G | 16325233 | 2 | *FOXP1* | Has a role in adipocyte differentiation, regulation of tissue and cell type-specific gene transcription during development and adulthood. | BIO6 | 0.277092 | (Liu *et al.*, 2019) |
| 12 | Gga_rs14046703_G | 17502782 | 2 | - |  | BIO11 | 0.2185 |  |
| 13 | GGaluGA090168_G | 521806 | 2 | *NRG2* | Involved primarily in the development of the nervous and cardiovascular systems. | Elevation | 0.483567 | (Mei and Nave, 2014) |
| 13 | Gga_rs15690310_A | 2914053 | 2 | *NPM1* | Involved in ribosome biogenesis, regulation of proliferation and growth suppression pathways. | BIO11 | 0.446624 | (Zarka *et al.*, 2020) |
| 13 | Gga_rs16697936_A | 8614002 | 2 | - |  | BIO11 | 0.374222 |  |
| 13 | Gga_rs15697934_G | 11349617 | 2 | *SGCD* | Involved in cardiomyopathy and muscular dystrophy. | BIO11 | 0.299999 | (Li *et al.*, 2022) |
| 13 | Gga_rs14061794_A | 13265234 | 2 | - |  | Soil clay content | 0.201973 |  |
| 13 | GGaluGA096906_C | 15134755 | 2 | *ENSGALG00010017293* |  | BIO11 | 0.28469 |  |
| 13 | GGaluGA097376_A | 16276058 | 2 | *ENSGALG00010016127* |  | BIO11 | 0.26367 |  |
| 14 | GGaluGA100538_A | 4148009 | 2 |  |  | BIO11 | 0.323332 |  |
| 14 | Gga_rs14075775_C | 7452309 | 2 | *UNKL* | Has a role in protein, Zink ion, and metal ion binding. | Soil clay content | 0.185774 | (Iso-Touru *et al.*, 2016) |
| 14 | GGaluGA102883_G | 8403962 | 2 | - |  | BIO11 | 0.213389 |  |
| 15 | Gga_rs15762378_A | 1219433 | 2 | *ARVCF* | Has functions related to adherents junctions, which are cadherin-based adhesive structures that connect actin filaments between neighbouring cells. | Soil clay content | 0.197845 | (Carnahan *et al.*, 2010) |
| 15 | GGaluGA108657_G | 7014165 | 2 | *MYO18B* | Associated with biological processes important for lipid metabolism and the development of skeletal muscle. | Soil slit content | 0.436453 | (Zhang *et al.*, 2017) |
| 15 | Gga_rs14091453_A | 7151427 | 2 | - |  | BIO11 | 0.186947 |  |
| 15 | Gga_rs14092446_C | 8127457 | 2 | *DDTL* | DDTL protein is scare, DDTL transcripts were found in many different types of tissues, with the highest levels seen in the liver, kidney, and adipose tissue. | BIO11 | 0.31617 | (Illescas *et al.*, 2020) |
| 15 | Gga_rs15026369_G | 12318502 | 2 | - |  | BIO11 | 0.418278 |  |
| 17 | Gga_rs14104760_A | 1164757 | 2 | - |  | Elevation | 0.251052 |  |
| 17 | GGaluGA112977_G | 2210126 | 2 | *LRSAM1* | Is associated with muscle disorder. | BIO11 | 0.331961 | (Pampouille *et al.*, 2018; Allais *et al.*, 2019) |
| 17 | GGaluGA113385_A | 2791572 | 2 | *CACNA1B* | Is involved in calcium channel activity. | BIO6 | 0.25633 | (Hu *et al.*, 2021) |
| 17 | Gga_rs14102617_A | 3288244 | 2 | *TNFSF8* | Contributes significantly to the initiation of apoptosis and also play critical roles in the immune response to Salmonella enteritis’s inoculation | Soil clay content | 0.285502 | (Wu *et al.*, 2015; Tohidi, Javanmard and Idris, 2018) |
| 17 | Gga_rs14101785_C | 4822703 | 2 | - |  | BIO11 | 0.47639 |  |
| 17 | Gga_rs13744398_A | 5933197 | 2 | *ZER1* | Is found to be associated with body mass index. | BIO11 | 0.222147 | (Iranzo-Tatay *et al.*, 2022) |
| 17 | GGaluGA115485_A | 6861230 | 2 | *RAPGEF1* | It plays a significant function in reproduction. | BIO6 | 0.29716 | (Azmal *et al.*, 2019) |
| 17 | Gga_rs13661747_G | 8386852 | 2 | - |  | BIO11 | 0.411003 |  |
| 17 | GGaluGA117147_A | 9307583 | 2 | *NDUFA8* | Plays a critical role in the transfer of electrons from NADH to the respiratory chain. | BIO6 | 0.381171 | (Lindholm *et al.*, 2014) |
| 17 | Gga_rs15789928_G | 9928790 | 2 | - |  | BIO11 | 0.381238 |  |
| 18 | Gga_rs15810909_G | 1051164 | 2 | *MYH1B* | Regulates muscle contraction, and plays an important role in skeletal muscle development. | BIO11 | 0.296162 | (Tajsharghi *et al.*, 2008; Bateman *et al.*, 2023) |
| 18 | GGaluGA118240_G | 1485545 | 2 | *MYOCD* | Associated with muscle development and muscle fibre regeneration and repair mechanisms. | BIO6 | 0.371973 | (Gu *et al.*, 2022; Bateman *et al.*, 2023) |
| 18 | Gga_rs15039004_A | 4155582 | 2 | *OGFOD3* | Identified to be associated with dyslipidaemia. | BIO11 | 0.377136 | (England *et al.*, 2017) |
| 19 | GGaluGA125678_A | 3310846 | 2 | *CLIP2* | Involved in neuronal migration/maturation. | BIO6 | 0.331196 | (Jabbi *et al.*, 2012) |
| 19 | Gga_rs15046418_A | 3565397 | 2 | - |  | BIO11 | 0.358099 |  |
| 20 | GGaluGA174700_G | 637585 | 2 | - |  | BIO8 | 0.219637 |  |
| 20 | GGaluGA178527_A | 7512082 | 2 | *CDH4* | Involved in diverse cell type sorting and potentially playing a crucial role in retinal development. | BIO11 | 0.216208 | (Bateman *et al.*, 2023) |
| 20 | GGaluGA178919_G | 8102870 | 2 | *MTG2* | Plays a role in the regulation of the mitochondrial ribosome assembly and of translational activity. | BIO11 | 0.369318 | (Bateman *et al.*, 2023) |
| 20 | Gga_rs14278862_A | 10874807 | 2 | - |  | BIO11 | 0.467704 |  |
| 21 | Gga_rs14281159_C | 365994 |  | *CAMTA1* | Involved in neuromuscular process controlling balance, heterocycle metabolic process, and positive regulation of transcription. | BIO6 | 0.335486 | (Marchesi *et al.*, 2021) |
| 21 | Gga_rs14281575_G | 873221 | 2 | - |  | Elevation | 0.260824 |  |
| 21 | Gga_rs15181561_A | 2128180 | 2 | *SSU72* | Involved in the C-terminal domain of RNA polymerase II dephosphorylation, RNA processing, and termination. | Elevation | 0.208739 | (Bateman *et al.*, 2023) |
| 21 | Gga_rs16178648_A | 2135314 | 2 | - |  | BIO11 | 0.3405 |  |
| 21 | GGaluGA183387_G | 2736349 | 2 | *AGRN* | Function in neurons is highly regulated by alternative splicing, glycan binding and proteolytic processing. Modulates calcium ion homeostasis in neurons, specifically by inducing an increase in cytoplasmic calcium ions. | BIO6 | 0.417864 | (Bateman *et al.*, 2023) |
| 22 | GGaluGA186811_G | 3783822 | 2 | *LRRTM4* | Involved in synapse development and maintenance of the nervous system. | BIO11 | 0.282366 | (Laurén *et al.*, 2003) |
| 23 | Gga_rs14289153_G | 2596404 |  | *YTHDF2* | Modulate mRNA metabolism, including mRNA maturation, translation, and degradation. | BIO6 | 0.347778 | (Zhao, Roundtree and He, 2017) |
| 23 | GGaluGA188346_A | 2889994 | 2 | - |  | BIO6 | 0.437524 |  |
| 23 | GGaluGA189562_A | 4841599 | 2 | *GJB5* | May aid in the transmission of signalling molecules within tissues. | BIO11 | 0.530405 | (Frankenberg *et al.*, 2007) |
| 24 | GGaluGA190726_G | 1174308 | 2 | - |  | BIO6 | 0.41518 |  |
| 24 | GGaluGA191239_G | 1969360 |  | *NTM* | It's associated with the control of oviduct development and differentiation in chickens. | BIO11 | 0.219049 | (Cho *et al.*, 2021) |
| 24 | Gga_rs14294033_G | 2317886 | 2 | *MED12* | An important regulator of hematopoietic stem cell homeostasis. | BIO6 | 0.382408 | (Zhang *et al.*, 2019) |
| 24 | Gga_rs15215725_A | 2374076 | 2 | *OPCML* | Found to be associated with body weight and growth rate. | BIO11 | 0.477885 | (Gu *et al.*, 2011) |
| 24 | GGaluGA191552_G | 2434624 | 2 | - |  | Soil clay content | 0.16693 |  |
| 24 | Gga_rs15216173_A | 2499086 | 2 | *IGSF9B* | Is involved in cell adhesion at synapses. | BIO11 | 0.383857 | (Bateman *et al.*, 2023) |
| 24 | Gga_rs16196837_A | 3705029 | 2 | *GRIK4* | Involved in the function of the central nervous system and has a role in excitatory synaptic transmission. | BIO8 | 0.176183 | (Bateman *et al.*, 2023) |
| 24 | GGaluGA193073_A | 4724363 | 2 | - |  | BIO11 | 0.357711 |  |
| 24 | GGaluGA193703_G | 5878930 | 2 | *NCAM1* | Is a cell adhesion molecule involved in neuron-neuron adhesion, neurite fasciculation, and outgrowth of neurites. | BIO11 | 0.328713 | (Bateman *et al.*, 2023) |
| 24 | GGaluGA193743_G | 5939657 | 2 | *ENSGALG00010026134* |  | BIO6 | 0.353195 |  |
| 26 | GGaluGA195343_A | 983689 | 2 | - |  | BIO11 | 0.381825 |  |
| 26 | Gga_rs14298585_A | 2077663 | 2 | *DSTYK* | Involved in induction of apoptosis. | BIO6 | 0.380363 | (Becic *et al.*, 2018) |
| 26 | GGaluGA196968_G | 3192081 | 2 | *TRAF3IP3* | Is expressed in the immune system, where it aids in cell maturation, tissue development, and immune response. | BIO6 | 0.39797 | (Li *et al.*, 2018) |
| 0 | GGaluGA345091_C | 0 | 3 |  |  | Soil organic carbon | 0.336306 |  |
