# Supplementary material for: The risk status, signatures of adaptation, and environmental suitability of village-based indigenous chickens from certain regions of Limpopo and KwaZulu-Natal provinces of South Africa
Source: Front Genet. 2024 Dec 18;15:1450939. doi: 10.3389/fgene.2024.1450939 (PMC11688331; doi:10.3389/fgene.2024.1450939)
Supplement: Supplementary file 3 [file DataSheet3.pdf]

Supplementary material

Table 3: Average r^2 values per chromosomes of conservations flock

Chr	Naked Neck	Potchefstroom Koekoek	Venda	Ovambo	New Hampshire	White Leghorn	White Plymouth Rock
1	0.12±0.15	0.09±0.11	0.13±0.17	0.14±0.17	0.22±0.24	0.20±0.23	0.17±0.19
2	0.11±0.14	0.08±0.11	0.13±0.17	0.14±0.17	0.23±0.24	0.19±0.22	0.17±0.19
3	0.12±0.16	0.07±0.10	0.13±0.17	0.14±0.16	0.21±0.24	0.20±0.23	0.16±0.19
4	0.11±0.14	0.08±0.12	0.13±0.17	0.14±0.17	0.26±0.27	0.18±0.21	0.15±0.18
5	0.11±0.16	0.08±0.11	0.12±0.15	0.14±0.16	0.21±0.23	0.18±0.21	0.16±0.19
6	0.10±0.13	0.08±0.10	0.12±0.16	0.14±0.17	0.22±0.24	0.17±0.21	0.15±0.18
7	0.10±0.14	0.08±0.10	0.13±0.17	0.14±0.16	0.21±0.23	0.18±0.21	0.15±0.18
8	0.11±0.14	0.08±0.11	0.12±0.16	0.14±0.16	0.22±0.24	0.18±0.22	0.15±0.18
9	0.09±0.12	0.08±0.10	0.09±0.14	0.13±0.16	0.23±0.24	0.17±0.21	0.15±0.18
10	0.11±0.14	0.08±0.11	0.12±0.18	0.13±0.17	0.23±0.23	0.18±0.22	0.14±0.18
11	0.13±0.16	0,09±0.12	0.11±0.14	0.14±0.17	0.19±0.21	0.19±0.21	0.14±0.19
12	0.11±0.15	0.08±0.10	0.17±0.21	0.15±0.18	0.19±0.21	0.17±0.20	0.14±0.17
13	0.11±0.14	0.08±0.11	0.11±0.15	0.15±0.17	0.17±0.20	0.16±0.19	0.15±0.18

14	0.11±0.14	0.07±0.1	0.10±0.15	0.14±0.16	0.20±0.22	0.22±0.26	0.15±0.18
15	0.11±0.15	0.08±0.11	0.12±0.16	0.15±0.18	0.21±0.24	0.18±0.21	0.13±0..16
16	0.19±0.23	0.14±0.20	0.27±0.42	0.26±0.25	0.34±0.29	0.5±0.3	0.17±0.22
17	0.10±0.14	0.08±0.20	0.10±0.14	0.13±0,16	0.17±0.20	0.17±0.21	0.14±0.17
18	0.09±0.13	0.08±0.11	0.11±0.14	0.14±0.17	0.20±0.22	0.16±0.21	0.15±0.17
19	0.09±0.13	0.07±0.09	0.09±0.13	0.13±0.15	0.23±0.23	0.17±0.22	0.15±0.17
20	0.09±0.13	0.08±0.11	0.10±0.15	0.13±0.15	0.22±0.24	0.16±0.20	0.16±20
21	0.09±0.12	0.08±0.10	0.12±0.16	0.13±0.17	0.18±0.21	0.18±0.23	0.15±0.19
22	0.10±0.14	0.09±0.12	0.12±0.19	0.14±0.17	0.23±0.24	0.18±0.20	0.15±0.18
23	0.11±0.15	0.07±0.10	0.11±0.15	0.14±0.16	0.15±0.18	0.17±0.21	0.16±0.19
24	0.10±0.13	0.07±0.09	0.09±0.14	0.14±0.16	0.19±0.22	0.20±0.23	0.14±0.17
25	0.09±0.14	0.08±0.11	0.10±0.14	0.13±0.15	0.19±0.23	0.20±0.22	0.14±0.18
26	0.11±0.15	0.08±0.11	0.09±0.14	0.13±0.15	0.17±0.20	0.15±0.20	0.17±0.20
27	0.11±0.14	0.08±0.11	0.10±0.14	0.13±0.16	0.20±0.22	-	-
28	0.09±0.13	0.07±0.10	0.12±0.16	0.12±0.15	0.17±0.21	-	-
